# Positive selection and heat‐response transcriptomes reveal adaptive features of the Brassicaceae desert model, *Anastatica hierochuntica*

**DOI:** 10.1111/nph.18411

**Published:** 2022-08-26

**Authors:** Gil Eshel, Nick Duppen, Guannan Wang, Dong‐Ha Oh, Yana Kazachkova, Pawel Herzyk, Anna Amtmann, Michal Gordon, Vered Chalifa‐Caspi, Michelle Arland Oscar, Shirli Bar‐David, Amy Marshall‐Colon, Maheshi Dassanayake, Simon Barak

**Affiliations:** ^1^ Albert Katz International School for Desert Studies Ben‐Gurion University of the Negev Sde Boqer Campus Midreshet Ben‐Gurion 8499000 Israel; ^2^ Department of Biological Sciences Louisiana State University Baton Rouge LA 70803 USA; ^3^ Institute of Molecular, Cell and Systems Biology, College of Medical, Veterinary and Life Sciences University of Glasgow Glasgow G12 8QQ UK; ^4^ Bioinformatics Core Facility, The National Institute for Biotechnology in the Negev Ben‐Gurion University of the Negev Beer‐Sheva 8410501 Israel; ^5^ Blaustein Center for Scientific Cooperation Ben‐Gurion University of the Negev Sde Boqer Campus Midreshet Ben‐Gurion 8499000 Israel; ^6^ Mitrani Department of Desert Ecology, Jacob Blaustein Institutes for Desert Research Ben‐Gurion University of the Negev Sde Boqer Campus Midreshet Ben‐Gurion 8499000 Israel; ^7^ Department of Plant Biology University of Illinois at Urbana‐Champaign Urbana IL 61801 USA; ^8^ French Associates' Institute for Agriculture and Biotechnology of Drylands, Jacob Blaustein Institutes for Desert Research Ben‐Gurion University of the Negev Sde Boqer Campus Midreshet Ben‐Gurion 8499000 Israel

**Keywords:** adaptation, *Anastatica hierochuntica*, Brassicaceae, desert species, extremophyte, heat stress, positive selection, transcriptome

## Abstract

- Plant adaptation to a desert environment and its endemic heat stress is poorly understood at the molecular level. The naturally heat‐tolerant Brassicaceae species *Anastatica hierochuntica* is an ideal extremophyte model to identify genetic adaptations that have evolved to allow plants to tolerate heat stress and thrive in deserts.
- We generated an *A. hierochuntica* reference transcriptome and identified extremophyte adaptations by comparing *Arabidopsis thaliana* and *A. hierochuntica* transcriptome responses to heat, and detecting positively selected genes in *A. hierochuntica*.
- The two species exhibit similar transcriptome adjustment in response to heat and the *A. hierochuntica* transcriptome does not exist in a constitutive heat ‘stress‐ready’ state. Furthermore, the *A. hierochuntica* global transcriptome as well as heat‐responsive orthologs, display a lower basal and higher heat‐induced expression than in *A. thaliana*. Genes positively selected in multiple extremophytes are associated with stomatal opening, nutrient acquisition, and UV‐B induced DNA repair while those unique to *A. hierochuntica* are consistent with its photoperiod‐insensitive, early‐flowering phenotype.
- We suggest that evolution of a flexible transcriptome confers the ability to quickly react to extreme diurnal temperature fluctuations characteristic of a desert environment while positive selection of genes involved in stress tolerance and early flowering could facilitate an opportunistic desert lifestyle.

## Introduction

Plant species inhabiting extreme environments – so‐called extremophytes – are able to thrive in the most inhospitable environments on Earth that are characterized by severe abiotic stresses. These stresses include drought and temperature extremes in deserts, intense cold in the Antarctic, and saline terrestrial and marine habitats (Amtmann, 2009; John *et al*., 2009; Dassanayake *et al*., 2010; Oh *et al*., 2012; Lawson *et al*., 2014; Farrant *et al*., 2015; Kazachkova *et al*., 2018; Oscar *et al*., 2018). Understanding the molecular mechanisms by which extremophytes adapt to their stressful environments could aid in identifying targets for molecular breeding to improve crop stress tolerance (Bressan *et al*., 2013; Shabala, 2013; Cheeseman, 2015). While tolerance to salt stress has been extensively investigated in halophytes that are adapted to highly saline environments (Flowers *et al*., 2015; Kazachkova *et al*., 2018; G. Wang *et al*., 2021), our understanding of molecular adaptations to stresses characteristic of desert habitats is still in its infancy (Granot *et al*., 2009; Yates *et al*., 2014; Oh *et al*., 2015; Obaid *et al*., 2016; Eshel *et al*., 2021; Wan *et al*., 2021). Yet, desert species could represent a treasure trove of molecular determinants that confer tolerance to multiple stresses such as drought, salinity, low soil nutrient levels, and heat stress. With global temperatures projected to continue rising (IPCC, 2021), plant adaptation to heat stress is of particular importance due to its negative effects on plant physiology, particularly at the reproductive stage, leading to severe reductions in yield (Mittler & Blumwald, 2010; Lesk *et al*., 2016; Chaturvedi *et al*., 2021; Y. Wang *et al*., 2021). Indeed, tolerance to heat stress in wheat at the reproductive stage has been identified as a key trait to increase yield potential under projected climate change (Stratonovitch & Semenov, 2015).

**Fig. 1:**
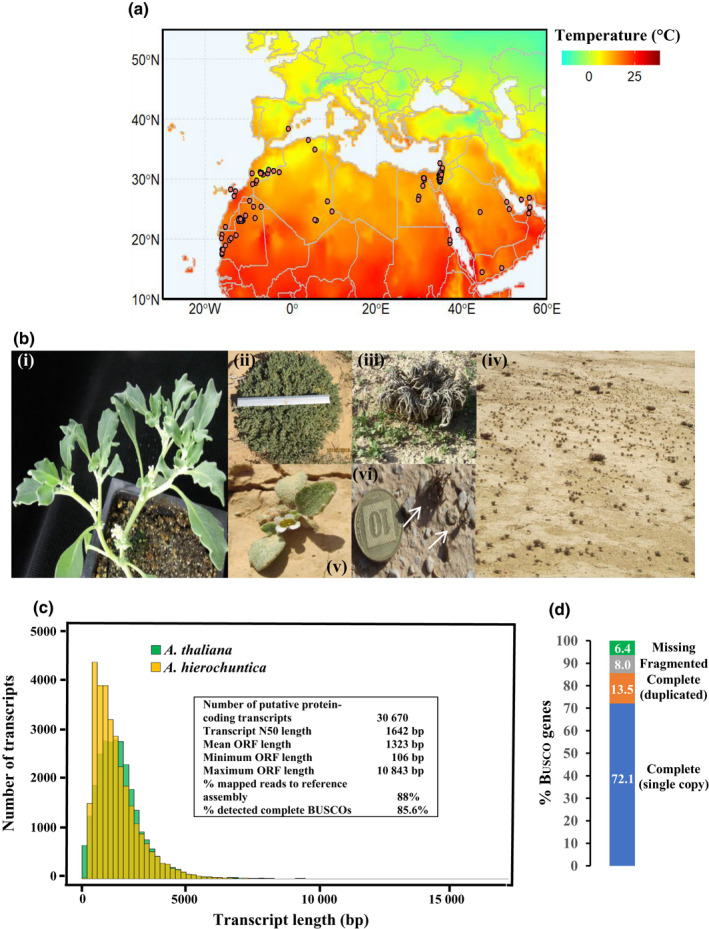
Geographic distribution of *Anastatica hierochuntica* and *de novo* reference transcriptome. (a) Geographic distribution data are based on *Anastatica* L. in the Global Biodiversity Information Facility (GBIF) database (GBIF.org [06 March 2021] GBIF Occurence Download https://doi.org/10.15468/dl.a52s3x). Average temperature data for this region are from 1948 to February 2021 acquired by the Physical Sciences Laboratory (Fan & van den Dool, 2008). (b) Laboratory‐grown and wild *A. hierochuntica* plants. Panels: (i) 40 d‐old laboratory‐grown plant (note the axillary inflorescence at each branch point); (ii) large mature plant from the Ovda valley population in the Negev desert. Ruler length = 30 cm; (iii) young seedlings growing near the dead mother plant from a Neot Smadar population in the Negev desert; (iv) a large population of *A. hierochuntica* in the Ovda valley with high variation in plant size due to spatial and temporal variations in water availability; (v) *A. hierochuntica* seedling already beginning to flower after producing four true leaves (Neot Smadar); (vi) two tiny dead plants (white arrows) from a population near the Dead Sea valley, having already dispersed their few seeds. A coin is included in the photograph to provide a visualization of scale. (c) Transcript length distribution and *A. hierochuntica* assembly descriptive statistics. (d) Assessment of reference transcriptome assembly completeness using the Benchmarking Universal Single‐Copy Orthologs (Busco) tool (Simão *et al*., 2015). The percentages of 1375 single‐copy genes, conserved among land plants, identified in the *A. hierochuntica* transcriptome are shown.

To gain insight into genetic adaptations that facilitate an extremophyte lifestyle, comparative physiological and molecular analyses of stress‐sensitive *Arabidopsis thaliana* and extremophyte Brassicaceae have proven to be a powerful approach (Kramer, 2010; Koenig & Weigel, 2015; Kazachkova *et al*., 2018). Indeed, these extremophyte Brassicaceae are becoming premier models for understanding plant adaptation to extreme environments with the development of genetic resources including chromosome‐level genome assemblies, natural accession collections, transformation protocols, and web resources ( http://extremeplants.org/) (Zhu *et al*., 2015; Kazachkova *et al*., 2018; Wang *et al*., 2019). Yet, an extremophyte Brassicaceae model that represents desert species has not hitherto been developed. Such a model could leverage the functional genomics knowledge that exists for *A. thaliana* thereby facilitating comparative analyses to reveal plant adaptations to the extreme desert environment. We have therefore been studying the *A. thaliana* relative, *Anastatica hierochuntica* L. (‘True Rose of Jericho’), a Saharo‐Arabian desert species (Fig. 1a) which also occupies the uppermost, driest zones of wadies or runnels of the Israeli Negev desert (Friedman & Stein, 1980; Friedman *et al*., 1981; Fig. 1b). This arid region has temperatures varying between −3.6°C and 46°C, an annual rainfall between 25 and 200 mm, and soil nitrate levels ranging from 0.4 to 4 mM (Gutterman, 2002; Ward, 2009; Eshel *et al*., 2017). We have demonstrated that *A. hierochuntica* is highly tolerant to heat and low soil nitrogen, and moderately tolerant to salt stress (Eshel *et al*., 2017). Moreover, the plant exhibits salt‐resilient photochemistry and displays constitutively higher levels of metabolites that have a role in scavenging reactive oxygen species, than *A. thaliana* (Eppel *et al*., 2014; Eshel *et al*., 2017).

In the current study, we assembled an *A. hierochuntica* reference transcriptome and used this resource for two approaches to identify adaptations to an arid environment in a desert annual extremophyte. In the first approach, comparative analysis of heat‐response transcriptomes revealed an *A. hierochuntica* transcriptome that is more reactive to heat than that of *A. thaliana*. In the second approach, positive selection analysis identified genes that could contribute to adaptation to extreme conditions in general, and those that could facilitate an opportunistic desert lifestyle.

## Materials and Methods

For all analyses, detailed methods are provided in Supporting Information Methods S1.

### Plant material and growth conditions

*A. hierochuntica* plants for *de novo* reference transcriptome sequencing were grown on Murashige–Skoog (Murashige & Skoog, 1962) plates for 5 d in the growth room (16 h (150 μmol photons m^−2^ s^−1^) : 8 h, light : dark; 22°C). For Illumina sequencing, plate‐grown seedlings were directly used. For Roche 454 sequencing, plate‐grown seedlings were transferred to pots containing *A. thaliana* soil growth medium (Weizmann Institute of Science, Rehovot, Israel) and kept in the growth room until plants developed four true fully‐expanded leaves. These plants were then treated as follows: (1) Control (field‐capacity, 22°C); (2) Drought stress (25% field capacity for 1 wk); (3) Salt shock (200 mM sodium chloride (NaCl) in the fertilizer solution), harvested after 1, 3 and 6 h; (4) Heat shock (45°C), harvested after 0.5, 1, and 2 h. Roots, shoots and flowers (where available) from these soil‐grown plants, were harvested separately and snap‐frozen in liquid nitrogen. In addition, mature seeds, were imbibed in water for 8.5 h.

**Fig. 2:**
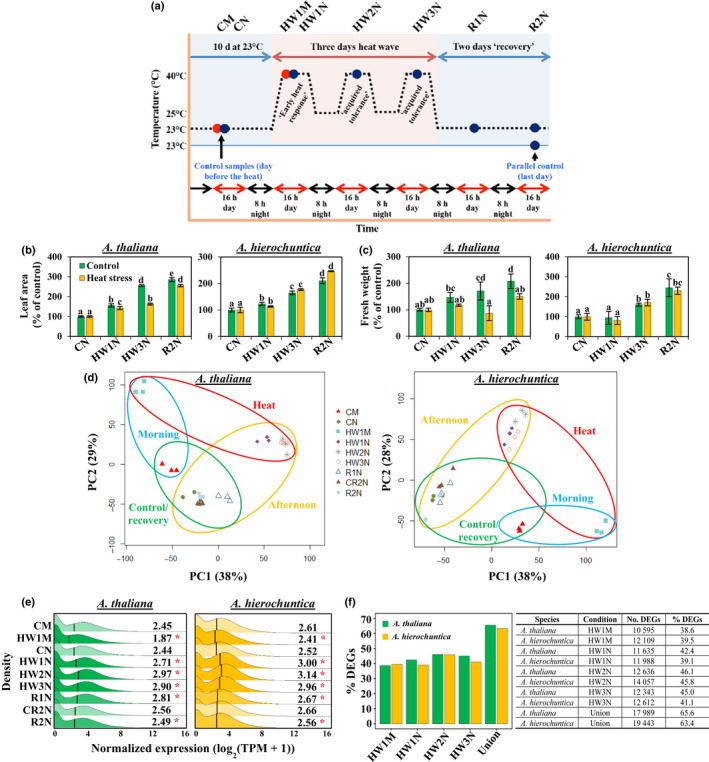
*Arabidopsis thaliana* and *Anastatica hierochuntica* exhibit similar transcriptome adjustment to heat stress. (a) Experimental design for *A. thaliana* and *A. hierochuntica* control and heat stress conditions. Control plants were harvested the day before the initiation of heat stress and on the last day of the experiment (indicated by vertical arrows) from a parallel 23°C control chamber. Red and blue circles represent samples harvested 1.5 h (morning) or 7 h (afternoon) respectively, after onset of light : heat. Each circle represents three independent experiments, each comprising six pooled plants. (b, c) Effect of heat stress on *A. thaliana* and *A. hierochuntica* leaf area (b) and fresh weight (c). Data are mean ± SD (*n* = 5) and are representative of two independent experiments. Letters above bars indicate significant difference at *P* < 0.05 (Tukey's HSD test). Blue shading, control conditions; Pink shading, heat conditions. (d) Principal component analysis (PCA) of *A. thaliana* and *A. hierochuntica* transcript levels. Each point represents one biological replicate and the three replicates for each condition are depicted with the same symbol. Symbols are explained in the legend box and refer to the experimental design shown in (a). (e) Comparison of the abundance of 27 416 and 30 670 protein‐coding *A. thaliana* and *A. hierochuntica* transcripts, respectively. Asterisks represent significant difference at *P* < 0.05 (Wilcoxon rank sum test) between the treatment compared to its respective control. Black vertical line within plots is median expression. (f) Percent of *A. thaliana* and *A. hierochuntica* differentially expressed genes (DEGs) in response to heat stress. In total, 17 989 *A. thaliana* and 19 443 *A. hierochuntica* genes were differentially expressed in response to heat stress in at least one condition (Supporting Information Dataset S2), and percent DEGs was calculated based on 27 416 and 30 670 protein‐coding genes for *A. thaliana* and *A. hierochuntica*, respectively. CM, control morning; CN, control afternoon; HW1M, heat wave 1 morning; HW1N, heat wave 1 afternoon; HW2N, heat wave 2 afternoon; HW3N, heat wave 3 afternoon; R1N, day 1 recovery from heat stress afternoon; CR2N, control plants parallel to the R2N time point afternoon; R2N, day 2 recovery from heat stress afternoon; Union, DEGs identified under either HW1M or HW1N or HW2N or HW3N.

For RNA‐sequencing (RNA‐Seq) heat stress experiments, *A. thaliana* and *A. hierochuntica* were grown on plates until cotyledons were fully expanded before transfer to pots containing *Arabidopsis* nitrogen‐less soil (Weizmann Institute of Science) and irrigation to field capacity with a custom‐made fertilizer solution (Methods S1). After 6 d in the growth room, uniform plants were transferred to two growth chambers (KBWF 720; Binder GmbH, Tuttlingen, Germany) (16 h : 8 h, light : dark; 23°C; 60% relative humidity; sunrise, 0.5 h at 100 μmol photons m^−2^ s^−1^; daytime, 250 μmol m^−2^ s^−1^; sunset, 0.5 h at 150 μmol photons m^−2^ s^−1^). Ten days after transfer to soil, heat treatment was initiated in one chamber comprising 3 d at 40°C : 25°C, day : night temperatures, followed by 2 d recovery at control conditions (Fig. 2a). The other chamber was kept as the control (23°C). For each condition, three biological replicates comprising six pooled plants per replicate (54 samples in total) were used for downstream analyses.

### Reference transcriptome and RNA‐Seq

RNA libraries were prepared using samples suitable for either the *A. hierochuntica* reference transcriptome or the RNA‐Seq transcriptome analysis:

1. For the reference transcriptome, equal amounts of total RNA from all samples (i.e. control, various stresses, tissues, time points, see section ‘Plant material and growth conditions’) were pooled and sent to the GenePool genomics facility at the University of Edinburgh, UK for 454 sequencing of a normalized complementary DNA (cDNA) library. In addition, total RNA from plate‐grown seedlings was sent to the Glasgow Polyomics Facility at the University of Glasgow, UK for Illumina sequencing. The reference transcriptome was assembled using a hybrid assembly approach that utilized both Illumina and 454 reads and was annotated based on public databases (Dataset S1; Methods S1).
2. For RNA‐Seq, total RNA was extracted from control and heat‐treated samples (Fig. 2a) and delivered to the Roy J. Carver Biotechnology Center, University of Illinois, Urbana‐Champaign, IL, USA for Illumina sequencing (Methods S1). Single‐end reads (100 bp) were uniquely mapped to *A. thaliana* TAIR 10 or the *A. hierochuntica* reference transcriptome using the Trinity align_and_estimate_abundance.pl script, which applies the Rsem program (Grabherr *et al*., 2011; Li & Dewey, 2011) with the Bowtie aligner.

Differentially expressed genes (DEGs) were identified using DESeq2 (Love *et al*., 2014; Methods S1). For raw read counts and DEGs identified in each species and for various functional groups, see Dataset S2.

**Fig. 3:**
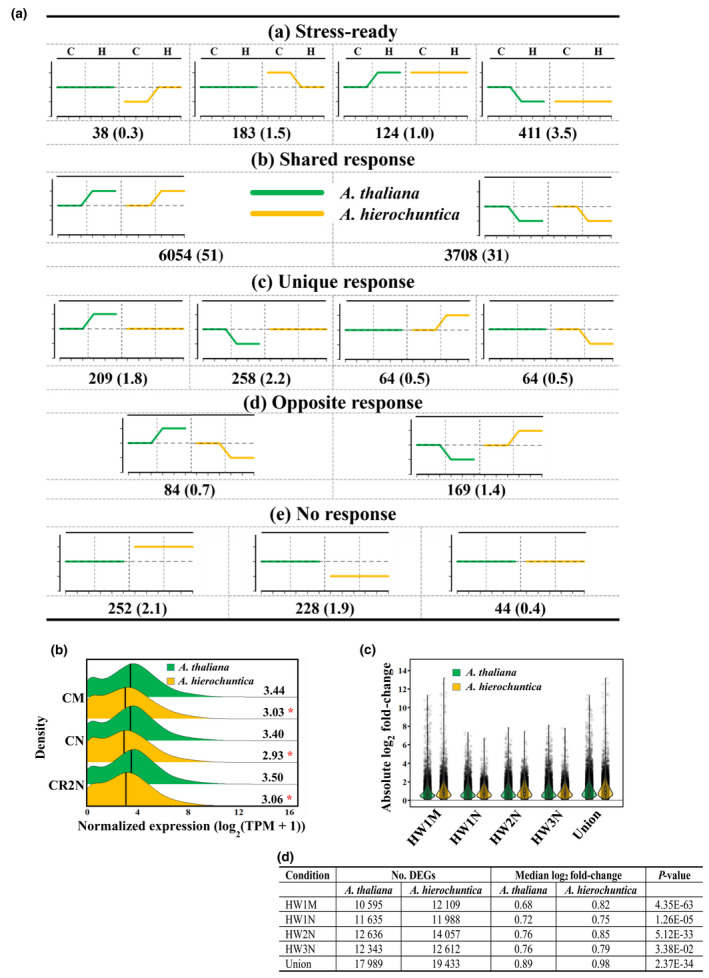
The *Anastatica hierochuntica* transcriptome does not exist in a ‘stress‐ready’ state but exhibits a lower basal expression and higher fold‐change expression than *Arabidopsis thaliana* in response to heat stress. (a) Modes of expression of ortholog pairs between *A. thaliana* and *A. hierochuntica* in response to heat stress. WGCNA followed by DESeq2 was used to assign orthologs to response modes (Supporting Information Fig. S3; Dataset S3). Differences in absolute transcripts levels were identified by comparing transcripts per kilobase million (TPM) minimum or maximum expression values (Student's *t*‐test, *P* ≤ 0.05). The green (*A. thaliana*) and orange (*A. hierochuntica*) lines indicate idealized expression patterns of the ortholog pairs in each species under control and heat conditions, compared to the *A. thaliana* control (dashed line). Ctrl, control; + heat, heat stress treatment; Numbers under graphs, no. of ortholog pairs assigned to each response mode; Numbers in parentheses, percent of ortholog pairs relative to the total number of orthologs (11 890) assigned to a response mode. (b) Transcript abundance of 17 962 *A. thaliana* and *A. hierochuntica* ortholog pairs under control conditions. (c) Combined violin and box plots showing absolute log_2_ fold‐changes of *A. thaliana* and *A. hierochuntica* differentially expressed genes (DEGs) in response to heat stress (Dataset S2). The median log_2_ fold‐change is shown as a black square inside each box plot. (d) Number of DEGs, median log_2_ fold‐change values and *P*‐values for (c). CM, control morning; CN, control afternoon; CR2N, control plants parallel to the R2N (day 2 recovery from heat stress afternoon) time point; HW1M, heat wave 1 morning; HW1N, heat wave 1 afternoon; HW2N, heat wave 2 afternoon; HW3N, heat wave 3 afternoon; Union, DEGs identified under either HW1M or HW1N or HW2N or HW3N. Asterisks represent significant difference at *P* < 0.05 (Wilcoxon rank sum test) between *A. thaliana* and *A. hierochuntica*.

Ortholog pairs (17 962; Methods S1) were assigned to the five idealized modes of expression in response to heat stress (Fig. 3a), using Weighted Gene Co‐expression Network Analysis (WGCNA; Langfelder & Horvath, 2008) to cluster normalized and quantified expression data into modules containing genes with similar expression profiles (Fig. S3; Dataset S3; Methods S1). For direct comparison of absolute orthologous transcript levels, transcripts per kilobase million (TPM) values of minimum or maximum expression were analyzed for statistically significant difference (*P* ≤ 0.05) with a Student *t*‐test.

### Phylogenomics and positive selection analysis

To identify orthologous genes among species and generate a maximum likelihood phylogenomic tree, we used coding sequences of the *A. hierochuntica* reference transcriptome and 16 sequenced Brassicaceae species (Methods S1) with the Agalma phylogenomics pipeline (Dunn *et al*., 2013).

To detect positive selection, we used the Branch‐Site model in the Paml v.4.8, Codeml program (Yang, 1997, 2007). Ortholog groups with sequence representation in at least two of the five extremophytes, were selected to ensure sufficient statistical power (Anisimova *et al*., 2001). The tested branch(s) were labeled (foreground), and the log likelihood of two models (M1a and M2a), were calculated for each ortholog group. A Likelihood Ratio Test was performed (with χ^2^ distribution), to identify genes with log likelihood values significantly different between the two models, indicative of deviation from neutral selection. Ortholog groups with a portion of sites in the foreground branches, that had an estimated dN : dS ratio > 1, were considered under positive selection. To account for multiplicity, a Benjamini–Yekutieli false discovery rate (FDR) correction (Benjamini & Yekutieli, 2001) was applied using the ‘qvalue’ R package, with a *q*‐value < 0.05 cut‐off for a gene to be considered as positively selected. Sites under positive selection were identified using the empirical Bayes approach with a posterior probability *P* > 0.95.

For each analysis, different branches on the tree were tested (labeled as foreground) compared with all other branches (background): (1) labeling the external branches of all five extremophyte species as the foreground (4723 ortholog groups); (2) labeling the *A. hierochuntica* external branch as the foreground (3093 ortholog groups); (3) labeling the *Eutrema salsugineum* external branch as the foreground (4457 ortholog groups); (4) labeling the *Schrenkiella parvula* external branch as the foreground (4369 ortholog groups); and (5) labeling the *A. thaliana* external branch as the foreground (5513 ortholog groups). *Arabidopsis thaliana* was considered as a control/comparator species sensitive to abiotic stresses (Kazachkova *et al*., 2018). The Venn diagram comparing positively selected genes (see later, Fig. 6b) was generated using the Venn online tool: http://bioinformatics.psb.ugent.be/webtools/Venn/.

Significant positively selected genes (PSGs) as well as all DEGs were tested for enriched gene ontology (GO) terms (Fisher's exact test, with a *q*‐value < 0.05 cut‐off) using agriGO (Du *et al*., 2010), where the *A. thaliana* genome served as background. Gomcl (Wang *et al*., 2020) was used to summarize nonredundant functional groups.

## Results

### *De novo* assembly and annotation of the *A. hierochuntica* reference transcriptome

To generate a high‐quality *A. hierochuntica* reference transcriptome that maximizes coverage of genes, we sequenced and assembled transcripts using RNA pooled from multiple plant organs, plants at different developmental stages, and under control, heat, drought and salinity stresses (Fig. S1; Methods S1). We identified 30 670 putative protein‐coding genes out of the high‐confidence 36 871 assembled transcripts (Fig. 1c; Methods S1), and the distribution of transcript lengths was similar to that of *A. thaliana* cDNAs (Fig. 1c). Detection of 93.6% Buscos indicated high completeness of the reference transcriptome (Fig. 1d; Simão *et al*., 2015), comparable to other *de novo* assembled Brassicaceae transcriptomes (Lopez *et al*., 2017). These data, together with 88% reads mapping back to the assembled transcriptome, indicate a high‐quality reference transcriptome appropriate for downstream analyses. Using sequence similarity to protein databases including NCBI, InterPro, and KEGG (Dataset S1), we annotated of 96% of our assembled transcripts to a known sequence in the reference databases (Methods S1).

### *A. thaliana* and *A. hierochuntica* global transcriptomes exhibit similar adjustment in response to heat stress

The transcriptomes of halophytic Brassicaceae models exist in a ‘stress‐ready’ state (Kazachkova *et al*., 2018; G. Wang *et al*., 2021). However, it is unknown whether a ‘stress‐ready’ transcriptome is the default for all extremophytes or whether plants evolving under different extreme environments exhibit alternate modes of adaptation. Therefore, to test whether a desert species exists in a ‘stress‐ready state’, we performed a comparative analysis of the *A. thaliana* and *A. hierochuntica* transcriptome response to heat stress in young plants at similar developmental stages. Israel Meteorological Service temperature data near *A. hierochuntica* populations during their growing season showed diurnal minimum : maximum night : day temperatures of *c*. 25°C and *c*. 40°C, respectively (Fig. S2). Thus, to simulate an ecologically relevant scenario with heat treatments that *A. thaliana* plants could also survive (Hayes *et al*., 2021), plants were exposed to similar three consecutive daily heat waves covering the early heat response and acquired heat tolerance phases (Lindquist, 1986; Hong & Vierling, 2000), with day : night temperatures of 40°C : 25°C followed by 2 d recovery at 23°C (Fig. 2a). To minimize shocks, temperatures were gradually ramped up and down at sunrise and sunset, respectively (Methods S1). Control plants were maintained at 23°C. Plants were harvested either in the morning (1.5 h after the onset of the light : heat period, red circles in Fig. 2a) or in the afternoon (7 h after the onset of the light : heat period, blue circles in Fig. 2a). Plants were well‐watered throughout the entire experiment to avoid any dehydration effects that could arise due to the heat treatment.

Heat stress had no significant effect on *A. hierochuntica* leaf area in contrast to *A. thaliana* where growth in leaf area was significantly retarded by heat stress although it had almost recovered to control levels, 2 d after the end of the heat treatment (Fig. 2b). *Arabidopsis thaliana* shoot fresh weight was also significantly reduced by heat stress but did not recuperate after 2 d recovery under control conditions while *A. hierochuntica* fresh weight was not affected by heat stress (Fig. 2c). These results illustrate that *A. hierochuntica* is highly tolerant to heat stress and confirm our previous *in vitro* experiments (Eshel *et al*., 2017).

Transcriptomes of both species under elevated temperature were clearly distinct from those in control conditions (Fig. 2d). The control and heat‐stressed samples harvested in the morning were positioned separately from the samples harvested in the afternoon, possibly due to differences in early vs late heat‐mediated gene expression or/and diurnal changes in gene expression. Transcriptomes of plants recovering from heat stress clustered near control noon samples suggesting that, overall, the transcriptomes return to pre‐stress conditions. Because both species underwent transcriptional adjustment in response to heat stress, we examined the median expression level across the whole transcriptome for each condition. Compared to their respective controls (CM, CN, CR2N), the median transcript abundance (and total abundance as depicted by the distribution) of both species decreased under heat stress in the morning samples, increased in response to heat treatments in the noon samples, and decreased during recovery (Fig. 2e). Furthermore, the percentage of DEGs (out of the total number of protein‐coding genes) was similar for both species under all heat conditions (Fig. 2f; Dataset S2). These data show that the *A. thaliana* and *A. hierochuntica* global transcriptomes adjust to heat stress with a similar magnitude.

### The *A. hierochuntica* heat‐response transcriptome does not exist in a ‘stress‐ready’ state

To test our contention that *A. hierochuntica* transcriptome is not ‘stress‐ready’, we used WGCNA to identify five types of idealized transcriptional response modes among the expression patterns of 17 962 orthologous pairs from each species (G. Wang *et al*., 2021; Fig. S3; Dataset S3): (1) ‘Stress‐ready’ where transcript level under control conditions in one species is equal to the ortholog transcript level under heat in the other species; (2) ‘Shared response’ where expression of both orthologs exhibit a similar response to heat (i.e. both upregulated or downregulated by heat); (3) ‘Unique response’ where expression of an ortholog exhibits a heat response specifically in one species but not in the other; (4) ‘Opposite response’ where expression of the ortholog in one species shows the opposite response in the other species; (5) ‘No response’ where expression of both orthologs does not respond to heat. Of the orthologs categorized within the five response modes, only 4.4% of the orthologs belonged to the ‘No response’ mode (Fig. 3a). The majority (82%) of orthologs displayed a shared response mode while about 5% exhibited a unique response and 2.1% showed an opposite response. Importantly, while 535 (4.5%) genes did exhibit a ‘stress‐ready’ mode in *A. hierochuntica*, we also detected 221 (1.9%) *A. thaliana* genes displaying a ‘stress‐ready’ mode. Taken together, our data showing that the global transcriptomes of both species adjust to heat stress with a similar magnitude and that they only exhibit a low proportion of ‘stress‐ready’ genes, do not support a globally ‘stress‐ready’ *A. hierochuntica* transcriptome.

### *A. hierochuntica* heat‐regulated genes display a higher fold‐change and/or lower basal expression compared to *A. thaliana*

**Fig. 4:**
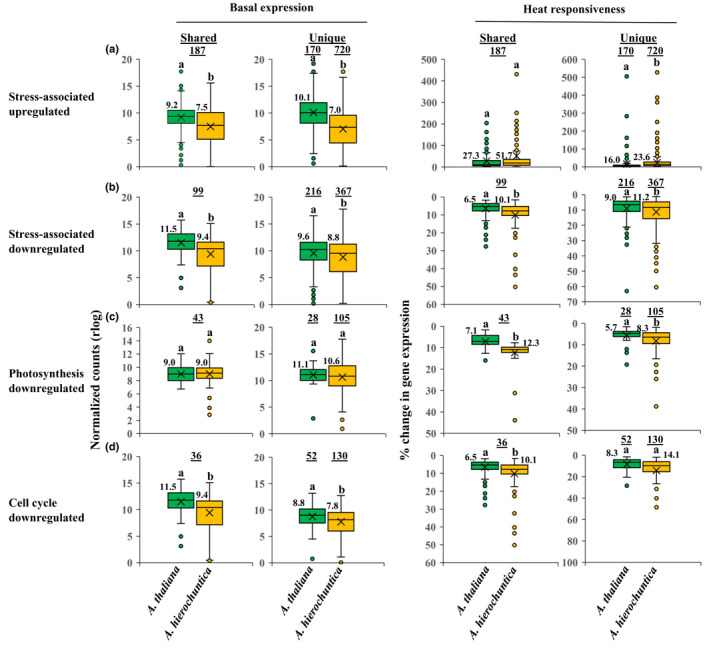
*Anastatica hierochuntica* shared‐ and unique‐expressed orthologs in specific functional groups display lower basal and greater heat‐mediated percent change in expression than in *Arabidopsis thaliana*. All genes used in this analysis possess a unique AGI code (putative *A. hierochuntica* orthologs were assigned *A. thaliana* AGI codes). Genes were chosen based on their association with gene ontology (GO) terms for their respective categories (Supporting Information Methods S1). Basal expression levels were based on CM (control morning) conditions. Percent change in expression from basal level was calculated based on the maximum rlog expression levels of upregulated genes (abiotic stress (a)) or minimum rlog expression levels of downregulated genes (abiotic stress (b), photosynthesis (c), cell cycle (d)) in response to heat stress over the three heat waves. Basal and percent change in expression values for all genes in each category are in Dataset S2. For box and whisker plots, the median (thick black line), the mean (cross below the median line) and interquartile range (IQR) of the observed differences are shown. Whiskers indicate the maximum/minimum range. Open circles correspond to extreme observations with values > 1.5 times the IQR. Underlined numbers above the circles indicate the number of shared or unique expressed genes. Letters above the circles indicate significant differences at *P* < 0.05 (Student's *t*‐test). Numbers next to boxes are median values.

Under control conditions, we observed that median basal expression of the *A. hierochuntica* transcriptome was significantly lower than in *A. thaliana* (Fig. 3b). Moreover, DEGs from the extremophyte displayed a greater heat‐mediated fold‐change in expression than *A. thaliana* DEGs (Fig. 3c,d) suggesting a more reactive heat‐response transcriptome. To support these findings, we compared orthologous expression of specific functional groups that exhibited either a shared or unique response mode to heat stress (Dataset S2). Orthologs associated with GO‐terms for abiotic stress whose expression displayed shared upregulation by heat exhibited an average lower basal expression in *A. hierochuntica* compared to *A. thaliana* and no significant difference in average percent induction of expression (Fig. 4a). Abiotic stress‐associated orthologs showing shared heat‐mediated downregulated expression displayed both a lower basal and higher percent reduction in expression in *A. hierochuntica* compared to *A. thaliana* (Fig. 4b). Similarly, both heat‐mediated upregulated/downregulated abiotic stress‐associated, unique‐expressed orthologs showed lower basal and higher percent induction/reduction in expression in the extremophyte (Fig. 4a,b).

Plants actively reduce their growth independently of photosynthesis, early in response to stress via a reduction in both cell size and cell elongation that can be linked to downregulation of cell cycle‐associated genes (Aguirrezabal *et al*., 2006; Skirycz *et al*., 2011; Kazachkova *et al*., 2013). Subsequently, expression of photosynthesis‐related genes is downregulated under stress (Rizhsky *et al*., 2002; X. Zhang *et al*., 2018; Huang *et al*., 2019). We observed that the majority of shared‐ and unique‐expressed orthologs associated with photosynthesis or the cell cycle were downregulated by heat stress in both species (Dataset S2). However, for both shared‐ and unique‐expressed orthologs associated with photosynthesis, *A. hierochuntica* exhibited a similar basal, but greater percent reduction in expression than *A. thaliana* (Fig. 4c). Orthologs encoding proteins involved in the cell cycle that possessed shared heat‐mediated downregulated expression showed a lower basal and higher percent reduction in expression in the extremophyte while unique‐expressed cell‐cycle orthologs exhibited lower basal expression in *A. hierochuntica* (Fig. 4d).

**Fig. 5:**
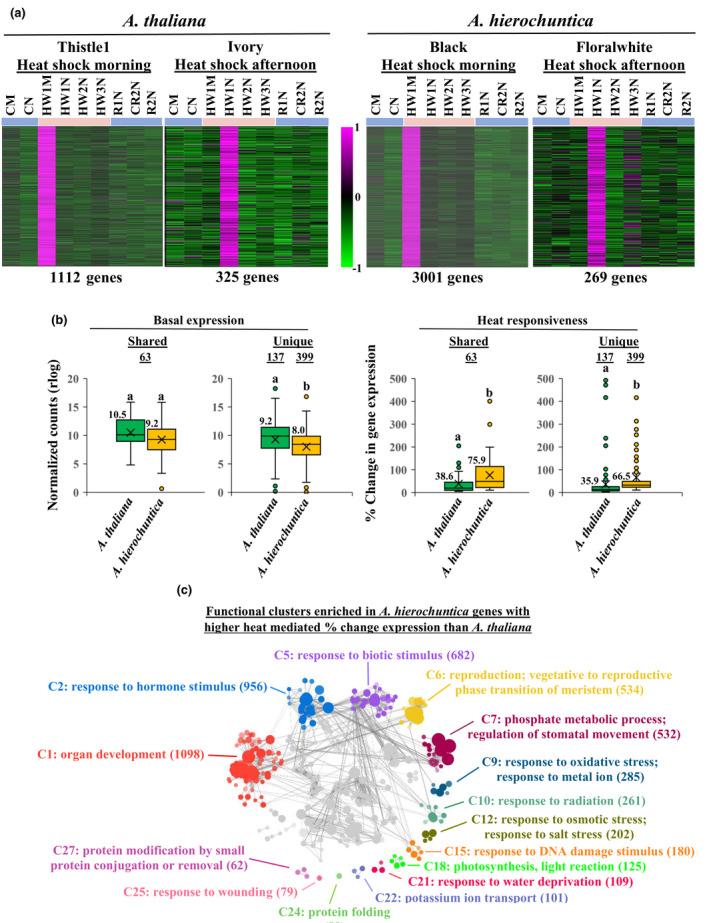
The *Anastatica hierochuntica* early heat response transcriptome displays lower basal and greater heat‐mediated percent change in expression than in *Arabidopsis thaliana*. (a) Expression profiles of *A. thaliana* (left two panels) and *A. hierochuntica* (right two panels) morning and afternoon early heat‐response modules. These modules were assigned standard color‐based names by WGCNA (e.g. Thistle, Ivory etc.; Supporting Information Fig. S3; Datasets S4–S8). Transcript levels were scaled to visualize patterns of expression. The relative intensity of gene expression (magenta, upregulated; green, downregulated) is shown in the scale bar. Gene expression in each condition represents the average of three biological replicates. The number of genes in each module is shown under the respective module. CM, control morning; CN, control afternoon; HW1M, heat wave 1 morning; HW1N, heat wave 1 afternoon; HW2N, heat wave 2 afternoon; HW3N, heat wave 3 afternoon; R1N, day 1 recovery from heat stress afternoon; CR2N, control plants parallel to the R2N time point afternoon; R2N, day 2 recovery from heat stress afternoon; Blue lines above heat map, control conditions; pink lines, heat conditions. (b) Expression of orthologs associated with abiotic stress gene ontology (GO) terms (Dataset S2; Methods S1) Underlined numbers above the circles indicate the number of shared‐ or unique‐expressed genes. Letters above the circles indicate significant differences at *P* < 0.05 (Student's *t*‐test). Numbers next to boxes are median values. (c) Functional clusters enriched among *A. hierochuntica* orthologous genes exhibiting a higher heat‐induced percent change in expression than *A. thaliana*. For full reactive gene list see Dataset S10. Clustering was performed with the Gomcl tool (https://github.com/Guannan‐Wang/GOMCL) (Wang *et al*., 2020; Methods S1) Clusters are colored differently and labeled with the representative functional term (Dataset S11). Each node represents a GO term and node size signifies the number of genes in the test set assigned to that functional term; the number of genes in each cluster is in parentheses. The shade of each node represents the *P*‐value assigned by the enrichment test (false discovery rate (FDR)‐adjusted *P* < 0.05) with darker shades indicating smaller *P*‐values. GO‐terms sharing > 50% of genes are connected by edges. Only selected clusters are highlighted, the rest are grayed out.

Using WGCNA to cluster genes with similar expression profiles over all conditions, we detected 22 *A. thaliana* and 21 *A. hierochuntica* co‐expression modules (Fig. S3). In both species, two modules covered early heat‐induced genes (1.5 h (morning) and 7 h (afternoon) after onset of heat stress) (Fig. 5a; Datasets S4–S8). The morning heat‐response modules of both species were enriched in GO biological terms such as ‘response to heat’, ‘response to high light intensity’ and ‘response to reactive oxygen species’ (Dataset S9; Methods S1), while the afternoon heat‐response modules were not enriched in any GO‐terms. Importantly, both shared‐ and unique‐expressed *A. hierochuntica* genes associated with GO terms for abiotic stress in the early heat‐response modules exhibited the same or lower basal expression, and higher heat‐mediated percent induction of expression than their *A. thaliana* orthologs (Fig. 5b).

To provide functional support for a more reactive *A. hierochuntica* heat‐response transcriptome, we identified 10 653 *A. hierochuntica* genes that exhibited significantly higher heat‐mediated percent change in expression than their *A. thaliana* orthologs (Dataset S10). These genes were enriched in biological processes related to abiotic stress including oxidative, water, and salt stresses, response to radiation (including genes involved in defense against UV light), and the response to DNA damage (Fig. 5c; Dataset S11). Additionally, the ‘protein folding’ gene list contained heat shock protein‐encoding genes.

To validate our gene expression comparisons, we showed (Fig. S4; Methods S1; Datasets S12, S13): (1) no significant difference between the species in the proportion of the top 10 most highly expressed genes out of the total transcripts sequenced across all treatments; (2) similar comparative basal expression results as observed with DESeq2, when we used a new between‐species Scale‐Based Normalization method (Zhou *et al*., 2019); (3) relative and quantitative PCR analysis confirmation of the RNA‐Seq fold‐change and basal gene expression patterns of selected genes.

Furthermore, we examined the basal expression of 15 orthologous housekeeping genes from both species and found that the average ratio of basal expression of *A. thaliana* to *A. hierochuntica* housekeeping genes was 1.0 ± 0.34 (Fig. S5). Thus, the average lower basal gene expression observed in *A. hierochuntica* compared to *A. thaliana* was not due to lower metabolic activity in the extremophyte.

### Brassicaceae extremophytes possess positively selected genes associated with surviving harsh environments

**Fig. 6:**
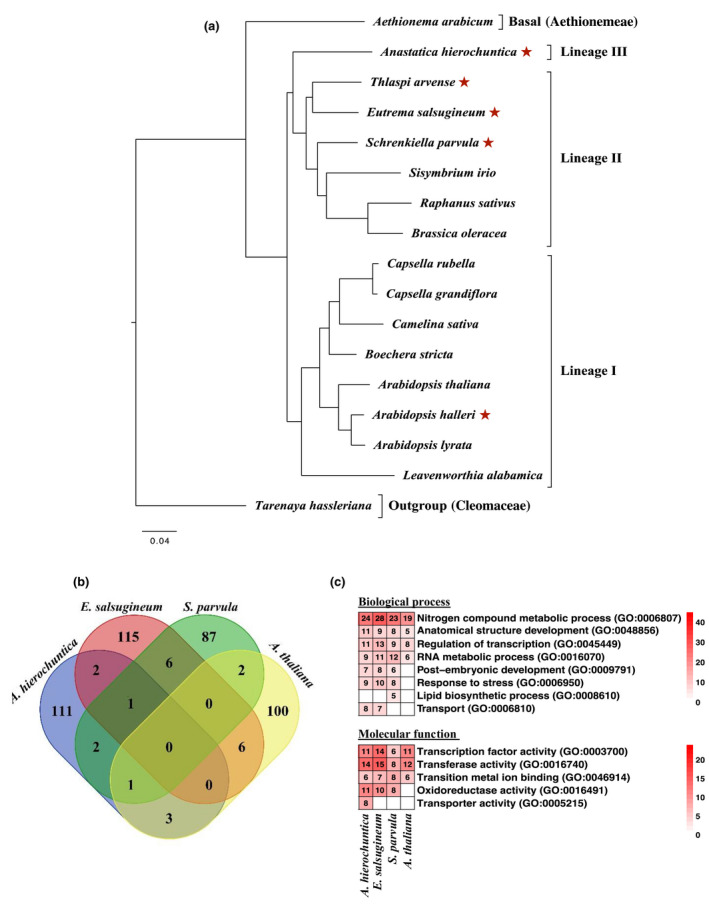
Phylogenomic and comparative positive selection analyses of *Anastatica hierochuntica* and other representative Brassicaceae genomes. (a) Maximum‐likelihood tree topology based on supermatrix analysis of 13 806 ortholog groups that contain an amino acid sequence from at least four taxa. All nodes are 100% supported by 100 rapid bootstrapping repeats. Red asterisks, extremophyte species. (b) Comparison of the number of positively selected genes (PSGs) among species. Positively selected genes in each species were identified using the Agalma‐Paml pipeline. (c) Comparative gene ontology (GO)‐term enrichment analysis of PSGs. The red color intensity corresponds to the number of PSGs assigned with that GO term (the numbers are indicated within the cells). Cells with a white color correspond to GO terms that were not significantly enriched. The *Arabidopsis thaliana* genome was used as the background gene set and significance (*q*‐value < 0.05) of enrichment was assessed via the Fisher's exact test. For the full list of enriched GO‐terms see Supporting Information Fig. S7; Datasets S20–S24).

As a second approach to identifying adaptations to an extremophyte lifestyle, in general, and to desert conditions in particular, we pinpointed PSGs that might be indicative of adaptive evolution of stress tolerance. We first used phylogenomics to infer evolutionary relationships between 16 Brassicaceae species including *A. hierochuntica* and representing all major lineages in this family (Dataset S14). *Tarenaya hassleriana* (Cleomaceae) was used as an outgroup. This led to a selection of 13 806 ortholog groups found in 17 taxa. The phylogenomic tree partitioned the species in concordance with their previously assigned lineages (LI, LII, and LIII), where *Aethionema arabicum* is considered to belong to a basal clade within the Brassicaceae (Fig. 6a; Franzke *et al*., 2011; Kiefer *et al*., 2014). *Anastatica hierochuntica* (Anastaticeae) was assigned to LIII (Franzke *et al*., 2011). It is important to note that *A. hierochuntica* is the single representative species used for LIII due to this lineage being sparsely represented in publicly available genomic databases unlike transcriptomes available for LI and LII species. Thus, to the best of our knowledge, we provide the first substantial genetic resource that enables exploration into adaptive traits that have evolved in a representative LIII species.

Comparative Brassicaceae transcriptome analysis has revealed that *A. hierochuntica* underwent a mesopolyploid event followed by diploidization (Mandakova *et al*., 2017). We therefore examined the orthologous groups for any bias towards *A. hierochuntica* using OrthoFinder (Emms & Kelly, 2019). *Anastatica hierochuntica* displayed a number of protein‐coding transcripts, and a number and percent of genes present in orthogroups, that were close to the average observed over all 17 species (Fig. S6). These data also suggest that the number of *A. hierochuntica* protein‐coding gene models in the curated reference transcriptome is not artificially inflated due to inclusion of a high proportion of alternatively‐spliced transcripts.

The tree contains five extremophyte species (Fig. 6a, red asterisks): the halophytes *E. salsugineum* and *S. parvula* (tolerant to high salinity and multiple other stresses; Kazachkova *et al*., 2018; G. Wang *et al*., 2021), *Thlaspi arvense* (freezing‐tolerant; Sharma *et al*., 2007), *A. hierochuntica* (heat‐, salt‐, low nitrogen‐tolerant; Eshel *et al*., 2017) and *Arabidopsis halleri* (heavy metal hyperaccumulator, semi‐alpine conditions; Hanikenne *et al*., 2008; Honjo & Kudoh, 2019). Therefore, to identify genes under common positive selective pressure in the extremophytes, we used the branch‐site model (Yang, 1997, 2007) to test the external branches (foreground) of the five extremophyte species against all the other branches (background). We then repeated this procedure to test for PSGs in three specific extremophytes – the well‐studied halophyte models, *E. salsugineum* and *S. parvula*, and *A. hierochuntica* – by labeling each species' external branch as the foreground. Overall, we identified 194, 120, 130 and 99 PSGs in the ‘all extremophyte species’, *A. hierochuntica*, *E. salsugineum*, and *S. parvula* runs, respectively (Datasets S15–S18). We also tested *A. thaliana* as an abiotic stress‐sensitive control, and identified 112 PSGs (Dataset S19).

While we could not detect a clear convergence in the use of common PSGs in the extremophytes (Fig. 6b), the functional attributes shared by those PSGs in each extremophyte exhibited convergence (Figs 6c, S7; Datasets S20–S24). Notably, orthologs associated with the GO‐term ‘response to stress’ (GO:0006950) were highly enriched in the extremophytes suggesting major selective pressure for stress tolerance imposed by their extreme environments.

**Fig. 7:**
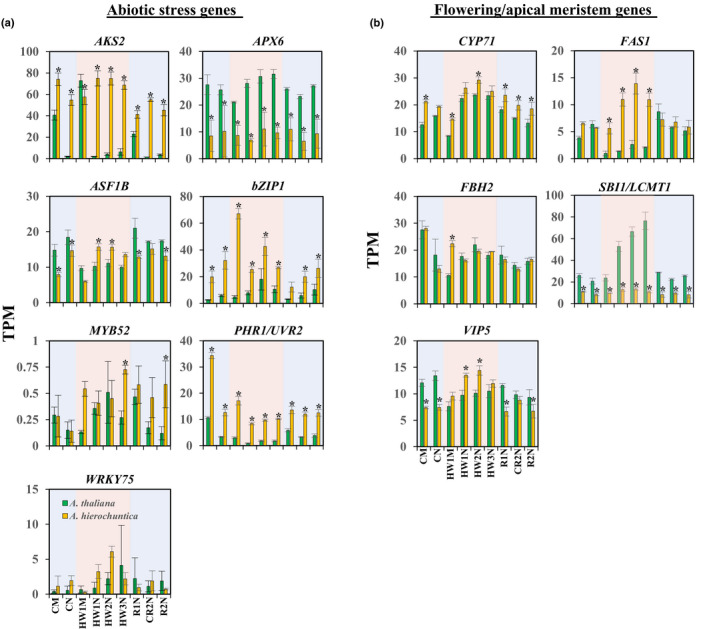
Expression of positively selected genes (PSGs) in response to heat stress. Gene expression was assessed by RNA‐sequencing transcriptome analysis of *Arabidopsis thaliana* and *Anastatica hierochuntica* plants grown under control conditions or exposed to heat stress (see Fig. 2a for experimental design). Expression is expressed as transcripts per kilobase million (TPM) normalized gene expression. (a) PSGs from the ‘all extremophyte species’ and *A. hierochuntica* analyses that are associated with abiotic stress responses (Table 1). (b) *A. hierochuntica* PSGs that function in photoperiodic flowering, regulation of meristems, and control of morphology (Table 1). Data are mean ± SD (*n* = 3) Asterisks indicate significant difference at *P* < 0.05 between *A. thaliana* and *A. hierochuntica* at the same time point and condition (Student's *t*‐test). CM, control morning; CN, control afternoon; HW1M, heat wave 1 morning; HW1N, heat wave 1 afternoon; HW2N, heat wave 2 afternoon; HW3N, heat wave 3 afternoon; R1N, day 1 recovery from heat stress afternoon; CR2N, control plants parallel to the R2N time point afternoon; R2N, day 2 recovery from heat stress afternoon; Blue shading, control conditions; Pink shading, heat conditions.

Positively selected genes from the ‘all extremophyte species’, supported association with adaptations to harsh environments. For instance, *AKS2*, *MYB52*, *WRKY75*, *ASF1B* and *PHR1*/*UVR2* that have known functions in abscisic acid (ABA) responses, phosphate starvation, heat stress, and UV‐B radiation stress, respectively (Table 1, and references cited therein), were among the PSGs in the extremophytes. Interestingly, the PSGs *bZIP1* (salt/drought tolerance, and nitrogen signaling) and *APX6* (reactive oxygen species‐scavenging) were unique to *A. hierochuntica* (Table 1), which is highly tolerant to low nitrogen and oxidative stresses, and moderately tolerant to salt stress (Eshel *et al*., 2017). Positively selected genes unique to *S. parvula* included *CAX11*/*CCX5* and *RAB28* that are involved in high‐affinity potassium ion (K^+^) uptake and sodium ion (Na^+^) transport, and lithium ion (Li^+^) toxicity, respectively (Table 1; Borrell *et al*., 2002; Zhang *et al*., 2011). The pinpointing of these two genes added validity to our positive selection analysis because the native soils of *S. parvula* contain highly toxic levels of Li^+^ and K^+^ (Helvaci *et al*., 2004; Ozfidan‐Konakci *et al*., 2016), and this species displays extreme tolerance to both Li^+^ and K^+^ toxicity (Oh *et al*., 2014; Pantha *et al*., 2021). In contrast to the extremophyte species, PSGs in *A. thaliana* were related to biotic stress responses (Table 1). Of the PSGs in the ‘all extremophyte species’ or *A. hierochuntica*‐specific sets, *AKS2*, *bZIP1* and *PHR1/UVR1* expression displayed significantly higher transcript levels in *A. hierochuntica* compared to *A. thaliana* whereas the expression of *APX6* exhibited lower transcript levels in *A. hierochuntica* (Fig. 7a).

**Table 1:** Positively selected genes^a^ with a potential role in adaptation to extreme environments.

Positively selected gene	Function	References
All extremophyte species (*Anastatica hierochuntica, Eutrema salsugineum, Schrenkiella parvula, Thlaspi arvense* and *Arabidopsis halleri*)		
*AKS2* (At1g05805)	Transcription factor (TF); facilitates stomatal opening, abscisic acid (ABA) response	Takahashi *et al*. (2013)
*ASF1B* (At5g38110)	Histone H3/H4 chaperone; repair of UV‐B‐induced DNA damage, basal and acquired thermotolerance	Lario *et al*. (2013); Nie *et al*. (2014); Weng *et al*. (2014)
*MYB52* (At1g17950)	TF; ABA response, drought tolerance, involved in the regulation of secondary wall formation, seed mucilage	Park *et al*. (2011); Cassan‐Wang *et al*. (2013); Shi *et al*. (2018)
*PHR1/UVR2* (At1g12370)	Photolyase enzyme; repair of UV‐B‐induced DNA damage	Ahmad *et al*. (1997); Jiang *et al*. (1997); Landry *et al*. (1997)
*WRKY75* (At5g13080)	TF; inorganic phosphate starvation, root development, GA‐mediated flowering, defense response	Devaiah *et al*. (2007); Velasco *et al*. (2016); Guo *et al*. (2017); L. Zhang *et al*. (2018)
*Anastatica hierochuntica*		
*APX6* (At4g32320)	Hydrogen peroxide‐scavenging enzyme; alleviation of reactive oxygen species damage	Chen *et al*. (2014)
*bZIP1* (At5g49450)	TF, light and nitrogen sensing, salt and drought tolerance	Obertello *et al*. (2010); Sun *et al*. (2012); Para *et al*. (2014)
*CYP71* (At3g44600)	Cyclophilin; silencing of homeotic genes; meristem development, interacts with FAS1 and the floral repressor LHP1	Li *et al*. (2007); Li & Luan (2011)
*FAS1* (At1g65470)	Subunit of CaF‐1; organization of apical meristems, cellular differentiation, DNA repair	Leyser & Furner (1992); Kaya *et al*. (2001); Hisanaga *et al*. (2013)
*FBH2* (At4g09180)	TF; photoperiodic flowering	Ito *et al*. (2012)
*SBI1/LCMT1* (At1g02100)	Leucine carboxylmethyltransferase; brassinosteroid signaling; flowering, stress responses	Di Rubbo *et al*. (2011); Wu *et al*. (2011); Creighton *et al*. (2017)
*VIP5* (At1g61040)	PAF1c component; activates floral repressors and photoperiodic pathway regulators. Regulation of nitrogen uptake	Oh *et al*. (2004); Yu & Michaels (2010); Crevillen & Dean (2011); Widiez *et al*. (2011); Lu *et al*. (2017)
*Eutrema salsugineum*		
*ATCES1/ACER* (At4g22330)	Alkaline ceramidase; sphingolipid homeostasis, disease resistance, salt tolerance	Wu *et al*. (2015)
*GRXS13* (At1g03850)	Glutaredoxin; chilling and photooxidative stress tolerance	Laporte *et al*. (2012); Hu *et al*. (2015)
*NCA1* (At3g54360)	Chaperone; regulates catalase 2 (reactive oxygen species‐scavenging enzyme) activity, salt, cold, high pH stresses	Li *et al*. (2015)
*PSRP2* (At3g52150)	Plastid‐specific ribosomal protein; RNA chaperone activity, negative regulator of seed germination under abiotic stress	Xu *et al*. (2013)
*SLK2* (At5g62090)	Transcriptional adaptor; embryogenesis, organ development, repression of stress‐responsive gene transcription	Bao *et al*. (2010); Lee *et al*. (2014); Shrestha *et al*. (2014)
*Schrenkiella parvula*		
*Atrab28* (At1g03120)	LEA protein; Li^+^ tolerance	Borrell *et al*. (2002)
*CAX11/CCX5* (At1g08960)	Cation calcium exchanger; K^+^ uptake, Na^+^ transport in yeast	Zhang *et al*. (2011)
*PER1* (At1g48130)	Peroxiredoxin; reactive oxygen species scavenging, enhances primary seed dormancy	Chen *et al*. (2020)
*Arabidopsis thaliana*		
*ATG6* (At3g61710)	AuTophGy‑related protein; autophagy, pathogen defense	Patel & Dinesh‐Kumar (2008)
*ATL2* (At3g16720)	RING‐H2 zinc‐finger protein; pathogen defense	Serrano & Guzmán (2004)
*ERDJ3B* (At3g62600)	ER‐localized DNAJ chaperone; anther development under heat stress, pathogen defense	Nekrasov *et al*. (2009); Yamamoto *et al*. (2020)
*RST1* (At3g27670)	ARM‐repeat protein; RNA exosome cofactor, vacuolar trafficking, cuticular wax production, embryo development, pathogen defense	Chen *et al*. (2005); Mang *et al*. (2009); Lange *et al*. (2019); Zhao *et al*. (2019)
*XLG2* (At4g34390)	Heterotrimeric G protein; pathogen defense	Liang *et al*. (2016)

Selected genes from five Codeml branch‐site model analyses are indicated based on their *A. thaliana* ortholog identifier.

^a^ For log‐likelihood values of the alternative and null models, log‐likelihood ratio tests and *P*‐values, see Supporting Information Datasets S15–S19.

Exclusively in *A. hierochuntica*, we identified, *CYP71*, *FAS1*, *FBH2*, *SBI1*/*LCMT1*, and *VIP5* as PSGs related to photoperiodic flowering, regulation of meristems, and control of morphology including shoot branching (Table 1). Furthermore, *AhFAS1* expression was highly upregulated by heat stress while *AtFAS1* expression was downregulated (Fig. 7b). *AhSBI1/LCMT1* expression was unaffected by heat stress whereas *AtSB1/LCMT1* expression was highly upregulated by heat. Moreover, *AhSBI1/LCMT1* transcript levels were lower than *AtSBI1/LCMT1* over all time points. Notably, genes involved in organ development and flowering time were more reactive to heat in *A. hierochuntica* than in *A. thaliana* (Fig. 5c). Considering that *A. hierochuntica* ontogeny is very different from *A. thaliana*, *E. salsugineum* and *S. parvula* – it exhibits a multi‐branched sympodial shoot structure supporting multiple axillary inflorescences that flower independent of day length (Fig. 1b; Gutterman, 1998; Eshel *et al*., 2017) – positive selection of these genes could indicate an important adaptation to the desert environment.

## Discussion

### The *A. hierochuntica* transcriptome does not exist in a heat ‘stress‐ready’ state and is more reactive to heat stress than *A. thaliana*

Our finding that *A. thaliana* and *A. hierochuntica* exhibit similar transcriptome adjustment in response to heat stress and during recovery (Fig. 2e,f) distinguishes *A. hierochuntica* from other extremophyte relatives. The extent of transcriptomic, proteomic and metabolic adjustment in response to ionic stress in the halophytes *E. salsugineum* and *S. parvula*, is much lower than in *A. thaliana* (Kazachkova *et al*., 2018; G. Wang *et al*., 2021). This lower adjustment reflects their ‘stress‐ready’ state whereby transcript and metabolite accumulation that is induced or repressed in *A. thaliana* in response to ionic stress, is constitutively high or low, respectively, in the halophytes. A ‘stress‐ready’ transcriptome is exemplified in *S. parvula* where basal expression of over 1000 ‘stress‐ready’ orthologs matches the post‐boron stress expression levels observed in *A. thaliana* (G. Wang *et al*., 2021). In contrast, the great majority of *A. hierochuntica* and *A. thaliana* orthologs exhibit a shared response mode (Fig. 3a). Furthermore, many stress‐related *A. hierochuntica* genes show lower basal and/or higher fold‐change gene expression compared to *A. thaliana* (Figs 3, 4, 5). Indeed, almost one‐third of *A. hierochuntica* genes display a higher heat‐mediated fold‐change in expression compared to *A. thaliana* and are enriched in abiotic stress‐associated functions (Fig. 5; Datasets S10, S11). Taken together, our findings support a paradigm whereby the *A. hierochuntica* transcriptome is more reactive to heat stress than *A. thaliana*.

The contrasting global transcriptome responses of *A. thaliana*, *E. salsugineum* and *A. hierochuntica* to stress also emphasize the importance of generation and maintenance of new *cis*‐acting elements in the adaptive evolution of plants to extreme habitats (Oh *et al*., 2014; He *et al*., 2021).

A possible reason for the divergent transcriptome responses between *A. hierochuntica* and its halophytic relatives relates to the type of stress each species encounters. *Eutrema salsugineum* and *S. parvula* habitats possess levels of ions such as Na^+^ and borate ion (BO_3_^3−^) that are toxic to most plant species and the two halophytes are constantly exposed to ionic stress throughout their life cycle. This situation might have led to the evolution of a stress‐associated transcriptome that is continuously ‘switched‐on’. Conversely, *A. hierochuntica* is generally exposed to heat stress later in its life cycle and on a diurnal basis thus favoring a more reactive transcriptome.

A second reason is that *A. hierochuntica* thrives in an environment with seasonal temperatures ranging from −3.6°C to 46.8°C (Eshel *et al*., 2017), with diurnal maximum variations exceeding 18°C (Israeli Meteorological Services). However, *E. salsugineum* can be found in locations such as China's Shandong peninsula where temperatures range from −5°C in the winter to 32°C in the summer and with diurnal temperature differences rarely exceeding 10°C (Guedes *et al*., 2015). Furthermore, diurnal temperature differences in the cold, spring‐growing period of *E. salsugineum* are likely more moderate than those experienced by *A. hierochuntica* during the warm Negev desert spring. Thus, evolution of a flexible transcriptome that confers a strong reaction to extreme diurnal temperature fluctuations could be advantageous for adaptation to a desert environment. Moreover, a transcriptome with globally lower basal expression levels would require less energy to be expended in the low nutrient desert environment.

Our results, notwithstanding, it is important to consider that we only analyzed transcriptome responses to heat stress alone to prevent masking of its effect by other stresses. In its natural desert habitat, *A. hierochuntica* can be exposed to multiple stresses including low relative humidity and drought, particularly in years of low precipitation. Combinations of stresses can cause unique morphophysiological states, gene sets and expression patterns that cannot be predicted from responses to single stresses (Zandalinas *et al*., 2021; Zandalinas & Mittler, 2022). For example, interactions between stresses can be synergistic, additive, or antagonistic whereby the effect of combined stresses is greater, equal to, or less, respectively, than the sum of effects of the respective single stresses (Shaar‐Moshe *et al*., 2017). Nevertheless, because a heat stress–drought stress combination generally leads to an additive or synergistic effect, it unlikely that this combination of stresses would reprogram the global ‘stress‐reactive’ *A. hierochuntica* transcriptome to one resembling a nonresponsive ‘stress‐ready’ transcriptome. However, stress combinations might cause a change in enriched functional GO‐terms in the *A. hierochuntica* ‘stress‐reactive’ gene set (Fig. 5c). Thus, it will be important in future studies to examine *A. hierochuntica* under different single and combined stresses to gain a better understanding of global transcriptome responses to conditions that mimic the natural desert habitat.

Another limitation of our study is that the *A. hierochuntica* global ‘stress‐reactive’ transcriptome response may not reflect that of the proteome and metabolome. However, evidence suggests that this is not the case because similar salt ‘stress‐ready’ transcriptomes, proteomes and metabolomes are observed in *E. salsugineum* compared to *A. thaliana* (Kazachkova *et al*., 2018).

### Brassicaceae extremophytes possess common PSGs that are indicative of adaptation to harsh environments

Extremophytes are present in all three Brassicaceae lineages (Fig. 6a; Franzke *et al*., 2011) illustrating that adaptation to stressful habitats has occurred independently, multiple times within the Brassicaceae and is indicative of convergent evolution (Fig. 6a; Birkeland *et al*., 2020). Consistent with this notion, we identified 194 PSGs across the five extremophyte species that could commonly contribute to plant adaptation to extreme environments. Other studies with extremophyte Brassicaceae have also detected PSGs that function in stress tolerance (Zhou *et al*., 2009; Jarvis *et al*., 2014; Birkeland *et al*., 2020). For instance, stress‐associated PSGs were detected in three Arctic Brassicaceae species (Birkeland *et al*., 2020). Similar to our findings (Figs 6b,c, S7) there was little overlap of PSGs between the Arctic extremophytes but considerable overlap in functional pathways. Taken together, these data do not support adaptive molecular convergence but rather indicate evolution of similar adaptations via distinct evolutionary pathways.

Among the PSGs across the five extremophyte species in the current study, we identified two genes encoding ABA‐responsive transcription factors (TFs), AKS2 and MYB52 (Table 1), illustrating the importance of the ABA response networks in adaptive evolution of stress tolerance (Xia *et al*., 2010; Fischer *et al*., 2011; Bondel *et al*., 2018). In particular, the basic helix–loop–helix (bHLH) TF, ABA‐RESPONSIVE KINASE SUBSTRATE 2 (AKS2) activates transcription of K^+^ channels in guard cells in an ABA‐dependent manner thereby enhancing stomatal opening (Takahashi *et al*., 2013). It is intriguing that a regulator of stomatal aperture has undergone positive selection across the extremophytes because alterations in stomatal aperture is a crucial early response to multiple abiotic stresses (Brugnoli & Lauteri, 1991; Chaves *et al*., 2009; Stepien & Johnson, 2009; Devireddy *et al*., 2020). Thus, positive selection of nonsynonymous amino acid changes in the coding region of *AKS2* (plus differences in heat‐mediated regulation of *A. thaliana* and *A. hierochuntica AKS2* expression (Fig. 7a)), suggest that this gene may have been naturally selected for survival in extreme environments.

*WRKY75* was also positively selected across the extremophytes (Table 1). In *A. thaliana*, this TF regulates the expression of several key phosphate starvation‐induced genes (Devaiah *et al*., 2007). Extremophytes often exist on soils with low inorganic phosphate (Pi) availability (Thompson *et al*., 2006; Holzapfel, 2008; Guevara *et al*., 2012). For instance, the *E. salsugineum* Yukon ecotype grows in the low Pi soils of the Yukon region in Canada (Guevara *et al*., 2012) and is highly tolerant to Pi deficiency compared to *A. thaliana*. This tolerance is associated with higher basal expression of several Pi starvation genes including *WRKY75* (Velasco *et al*., 2016). Both *E. salsugineum* and *A. hierochuntica* exhibit significantly higher basal levels of Pi than *A. thaliana* (Gong *et al*., 2005; Kazachkova *et al*., 2013; Velasco *et al*., 2016; Eshel *et al*., 2017). Thus, positive selection of *WRKY75* across the five extremophyte plants, and differential expression of *A. thaliana* and *E. salsugineum WRKY75* suggests that selection for more efficient extraction of soil Pi is a common evolutionary adaptation to extreme environments.

Extremophytes are often exposed to UV‐B radiation that can cause direct damage to DNA (Kimura & Sakaguchi, 2006). *PHOTOLYASE1*/*UV‐RESISTANCE2* (*PHR1*/*UVR2*) and *ANTI‐SILENCING FUNCTION 1B* (*ASF1B*) that are crucial for repairing UV‐B‐induced DNA damage were positively selected across the five extremophytes (Table 1; Ahmad *et al*., 1997; Jiang *et al*., 1997; Landry *et al*., 1997; Lario *et al*., 2013; Nie *et al*., 2014). *PHR1*/*UVR2* is also the major mechanism maintaining transgenerational genome stability in *A. thaliana* continuously exposed to UV‐B (Willing *et al*., 2016) while *ASF1B* is also involved in the regulation of basal and acquired thermotolerance (Weng *et al*., 2014). Additionally, *PHR1*/*UVR2* displays higher basal expression in *A. hierochuntica* compared to *A. thaliana*, and while heat leads to downregulation of the gene in both species, expression is reduced to a lesser extent in the extremophyte (Fig. 7a).

Collectively then, our data suggest common selective pressures in extremophyte plants that target key components in stomatal opening, nutrient acquisition, and UV‐B‐induced DNA repair. On the other hand, we found that *A. thaliana* PSGs were principally involved in defense against pathogens (Table 1). This supports the hypothesis that because *A. thaliana* evolved in temperate regions where pathogen density is relatively high compared to extremophyte habitats, it encountered greater evolutionary pressures for adaptation to biotic stresses (Oh *et al*., 2014).

### *A. hierochuntica* PSGs suggest adaptive evolution for an opportunistic desert lifestyle

We pinpointed a number of PSGs specifically in *A. hierochuntica* indicating adaptation to the desert environment (Table 1; Fig. 7b). Intriguingly, several of these genes function in *A. thaliana* in the transition from vegetative to reproductive growth and meristem development: (1) *VERNALIZATION INDEPENDENCE 5* (*VIP5*) enhances transcription of the floral repressor *FLOWERING LOCUS C* (*FLC*) gene and other *MADS AFFECTING FLOWERING* (*MAF*) gene family members (Oh *et al*., 2004; Yu & Michaels, 2010; Crevillen & Dean, 2011; Lu *et al*., 2017) and *A. thaliana vip5* mutants exhibit early flowering; (2) FLOWERING BHLH 2 (FBH2) activates transcription of the *CONSTANS* gene, a central regulator of photoperiodic flowering. *FBH2* overexpression causes photoperiod‐independent early flowering (Ito *et al*., 2012); (3) *FASCIATA1* (*FAS1*) appears to function in the organization of shoot and root apical meristems, and in cellular differentiation (Kaya *et al*., 2001; Exner *et al*., 2006). Mutations in *fas1* cause stem fasciation, abnormal leaf and flower morphology, and defects in the organization of apical meristems (Leyser & Furner, 1992; Kaya *et al*., 2001); (4) *CYP71* plays a critical role in regulating meristem development, including the floral meristem (Li *et al*., 2007). Furthermore, CYP71 physically interacts with FAS1 thereby targeting FAS1 to the *KNAT1* locus (Li & Luan, 2011). KNAT1 is essential for maintenance of apical meristems (Hake *et al*., 2004). In addition, CYP71 interacts with LIKE HETEROCHROMATIN PROTEIN 1 (LHP1), which is involved in repressing expression of flowering time and floral identity genes (Gaudin *et al*., 2001; Kotake *et al*., 2003). Thus, *lhp1* mutations cause strong early flowering; (5) *SUPPRESSOR OF BRI1 (SBI1)*/*LEUCINE CARBOXYLMETHYLTRANSFERASE (LCMT1)* regulates components of the brassinosteroid signaling pathway (Di Rubbo *et al*., 2011; Wu *et al*., 2011) and the *sbi1/lcmt* mutant is early flowering in both long and short days consistent with the role of brassinosteroids in flowering (Li & He, 2010; Nolan *et al*., 2020).

The discovery of positively selected flowering and meristem development genes specifically in *A. hierochuntica* is consistent with its very different developmental program compared to many other Brassicaceae including the additional four extremophyte plants included in our analysis. *A. hierochuntica* does not display the distinctive transition from the vegetative rosette leaf stage to the reproductive bolting stage, which is accelerated in long‐day conditions (Pouteau & Albertini, 2009; Song *et al*., 2013). Instead, regardless of photoperiod, the shoot repeatedly bifurcates from the four true‐leaf stage onwards, developing an axillary inflorescence at each branch point thereby leading to a multi‐branched shoot morphology (Fig. 1b, panel (i); Eshel *et al*., 2017). Most interestingly, mutation in the *A. thaliana FAS1* gene (whose *A. hierochuntica* ortholog is under positive selection) can induce stem bifurcation and enlargement (Leyser & Furner, 1992). The shoot bifurcation, multi‐branch, photoperiod‐insensitive, early flowering traits could maximize fitness in the unpredictable desert environment where plants need to ensure development of seeds but might not survive until a critical day length induces flowering. This idea is supported by our observations of *A. hierochuntica* populations in the Dead Sea valley of Israel, where tiny dead plants that have still managed to produce a few seeds can be seen alongside much larger plants presumably from a year with higher rainfall (Fig. 1b).

In conclusion, we have shown that *A. hierochuntica* possesses a more reactive heat‐response transcriptome, and stress‐related genes that have undergone positive selection. Genes that could be associated with its multi‐branch, early flowering phenotype also exhibit signatures of positive selection. Together, these evolutionary adaptations could allow survival in a hot desert environment with unpredictable precipitation. Our study furthermore provides rich gene sets that will facilitate comparative and functional genomics studies to reveal additional molecular mechanisms for plant tolerance to heat stress in a desert habitat.

## Author contributions

SB conceptualized and supervised the overall project; GE and ND performed the main analyses; GW contributed to transcriptome response assessment and performed the Gomcl analysis; GE, D‐HO, MG, MD and VC‐C contributed to assembly of the *A. hierochuntica* reference transcriptome. MD assisted with, and GE performed the positive selection analysis; YK and MAO contributed to plant growth, preparation of samples and transcriptome response validation; AA and PH designed, supervised and analyzed the Illumina RNA‐Seq experiment for the reference transcriptome at Glasgow Polyomics; AM‐C contributed to the WGCNA; GE, ND, GW, SB and MD contributed to data interpretation, GE, ND and SB wrote the article. MD, SB‐D and AA critically revised and approved the final manuscript. GE and ND contributed equally to this work.

## Supporting information


**Dataset S1** *Anastatica hierochuntica* transcriptome functional annotation.

**Dataset S2** *Arabidopsis thaliana* and *Anastatica hierochuntica* raw read data plus gene ontology‐terms, and differentially expressed genes.

**Dataset S3** Assignment of ortholog pairs modes of expression (log_2_ fold‐change data and WGCNA modules).
**Dataset S4** Early heat‐response modules (rlog expression data).
**Dataset S5** *Arabidopsis thaliana* early heat module (Thistle1) gene list.
**Dataset S6** *Arabidopsis thaliana* late heat module (Ivory) gene list.
**Dataset S7** *Anastatica hierochuntica* early heat module (Black) gene list.
**Dataset S8** *Anastatica hierochuntica* early heat module (Floralwhite) gene list.
**Dataset S9** Gene ontology‐term enrichment of *Arabidopsis thaliana* and *Anastatica hierochuntica* morning heat‐response modules.

**Dataset S10** Difference in percent change orthologous gene expression (based on TPM) between *Anastatica hierochuntica* and *Arabidopsis thaliana*.

**Dataset S11** Gene ontology‐term enrichment of genes that are more responsive to heat in *Anastatica hierochuntica* (see Fig. 5c).

**Dataset S12** One hundred and nine most conserved orthologs (≥ 98% query coverage, ≥ 99% nucleotide identity, ≥ Blast value of e‐100) between *Arabidopsis thaliana* and *Anastatica hierochuntica*.
**Dataset S13** PCR primers used in this study.

**Dataset S14** Species included in the custom Brassicaceae CDS database for the Brassicaceae phylogenomic analysis.
**Dataset S15** Codeml positive selected genes (*q* < 0.05) in the all extremophyte species run with the branch‐site model.
**Dataset S16** Codeml positive selected genes (*q* < 0.05) in *Anastatica hierochuntica* run with the branch‐site model.
**Dataset S17** Codeml positive selected genes (*q* < 0.05) in *Eutrema salsugineum* run with the branch‐site model.
**Dataset S18** Codeml positive selected genes (*q* < 0.05) in *Schrenkiella parvula* run with the branch‐site model.
**Dataset S19** Codeml positive selected genes (*q* < 0.05) in *Arabidopsis thaliana* run with the branch‐site model.
**Dataset S20** Gene ontology‐terms overrepresented in the all extremophyte species 194 positive selected genes.
**Dataset S21** Gene ontology‐terms overrepresented in *Anastatica hierochuntica* 120 positive selected genes.
**Dataset S22** Gene ontology‐terms overrepresented in *Eutrema salsugineum* 131 positive selected genes.
**Dataset S23** Gene ontology‐terms overrepresented in *Schrenkiella parvula* 99 positive selected genes.
**Dataset S24** Gene ontology‐terms overrepresented in *Arabidopsis thaliana* 112 positive selected genes.

**Fig. S1** Transcriptome sequencing and hybrid assembly workflow.
**Fig. S2** An example of diurnal temperatures over 3 d in the Dead Sea valley during April 2008.
**Fig. S3** Clustering dendrogram of module eigenvalues for *Arabidopsis thaliana* and *Anastatica hierochuntica* transcriptome profiles under heat stress conditions.
**Fig. S4** Validation of ‘between species’ RNA‐sequencing analysis.
**Fig. S5** Basal (control) expression of 15 orthologous *Arabidopsis thaliana* and *Anastatica hierochuntica* housekeeping genes.
**Fig. S6** Number of protein‐coding transcripts and comparative ortholog group composition for species used to detect positively selected genes.
**Fig. S7** Gene ontology‐term enrichment analysis of positively selected genes.
**Methods S1** Additional information regarding methods used in this study.
Please note: Wiley Blackwell are not responsible for the content or functionality of any Supporting Information supplied by the authors. Any queries (other than missing material) should be directed to the *New Phytologist* Central Office.

## Data Availability

Reference transcriptome and RNA‐Seq reads as well as the fully assembled transcriptome are openly available via the NCBI SRA and TSA databases under BioProject PRJNA731383. Other main data that supports the findings of this study are available in the main text and Supporting Information of this article.
