# Supplementary material for: Positive selection and heat‐response transcriptomes reveal adaptive features of the Brassicaceae desert model, Anastatica hierochuntica
Source: New Phytol. 2022 Aug 26;236(3):1006–26. doi: 10.1111/nph.18411 (PMC9804903; doi:10.1111/nph.18411)
Supplement: Supplementary file 8 — Fig. S1 Transcriptome sequencing and hybrid assembly workflow. Fig. S2 An example of diurnal temperatures over 3 d in the Dead Sea valley during April 2008. Fig. S3 Clustering dendrogram of module eigenvalues for Arabidopsis thaliana and Anastatica hierochuntica transcriptome profiles under heat stress conditions. Fig. S4 Validation of ‘between species’ RNA‐sequencing analysis. Fig. S5 Basal (control) expression of 15 orthologous Arabidopsis thaliana and Anastatica hierochuntica housekeeping genes. Fig. S6 Number of protein‐coding transcripts and comparative ortholog group composition for species used to detect positively selected genes. Fig. S7 Gene ontology‐term enrichment analysis of positively selected genes. Methods S1 Additional information regarding methods used in this study. Please note: Wiley Blackwell are not responsible for the content or functionality of any Supporting Information supplied by the authors. Any queries (other than missing material) should be directed to the New Phytologist Central Office. [file NPH-236-1006-s006.pdf]

New Phytologist

***New Phytologist* Supporting Information**

Article title: **Positive selection and heat-response transcriptomes reveal adaptive features of the Brassicaceae desert model, *Anastatica hierochuntica***

Authors: Gil Eshel, Nick Duppen, Guannan Wang, Dong-Ha Oh, Yana Kazachkova, Pawel Herzyk, Anna Amtmann, Michal Gordon, Vered Chalifa-Caspi, Michelle Arland Oscar, Shirli Bar-David, Amy Marshall-Colon, Maheshi Dassanayake and Simon Barak

Article acceptance date: *18 July 2022*

The following Supporting Information is available for this article:

Fig. S1 Transcriptome sequencing and hybrid assembly workflow.

Fig. S2 An example of diurnal temperatures over three days in the Dead Sea valley during April 2008.

Fig. S3 Clustering dendrogram of module eigenvalues for *A. thaliana* and *A. hierochuntica* transcriptome profiles under heat stress conditions.

Fig. S4 Validation of 'between species' RNA-seq analysis.

Fig. S5 Basal (control) expression of 15 orthologous *A. thaliana* and *A. hierochuntica* housekeeping genes.

Fig. S6 Number of protein-coding transcripts and comparative ortholog group composition for species used to detect positively selected genes.

Fig. S7 GO-term enrichment analysis of positively selected genes.

Methods S1 Additional information regarding methods used in this study.

Fig. S1 Transcriptome sequencing and hybrid assembly workflow. RNA was extracted from plate-grown *A. hierochuntica* seedlings under control conditions (Illumina sequencing), or from soil-grown plants under control or various abiotic stress conditions, and imbibed seeds (454 sequencing). To assemble both short- and long-read sequences together, a hybrid approach was taken. The Illumnia reads were first assembled into long contig sequences, using the Trinity assembler, and then shredded into 700 bp long, overlapping (at least 200 bp overlap) pseudo-reads that were reassembled together with the 454 reads using the Newbler assembler.

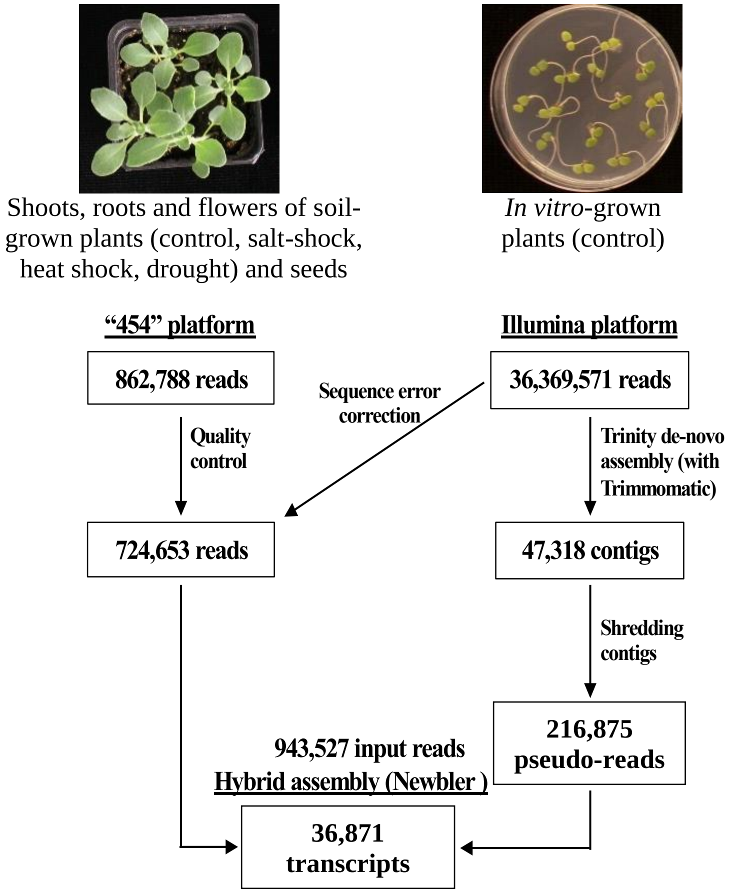

Fig. S2 An example of diurnal temperatures over three days in the Dead Sea valley during April 2008. Data were obtained from the Israel Meteorological Service (http://www.ims.gov.il/IMSEng).

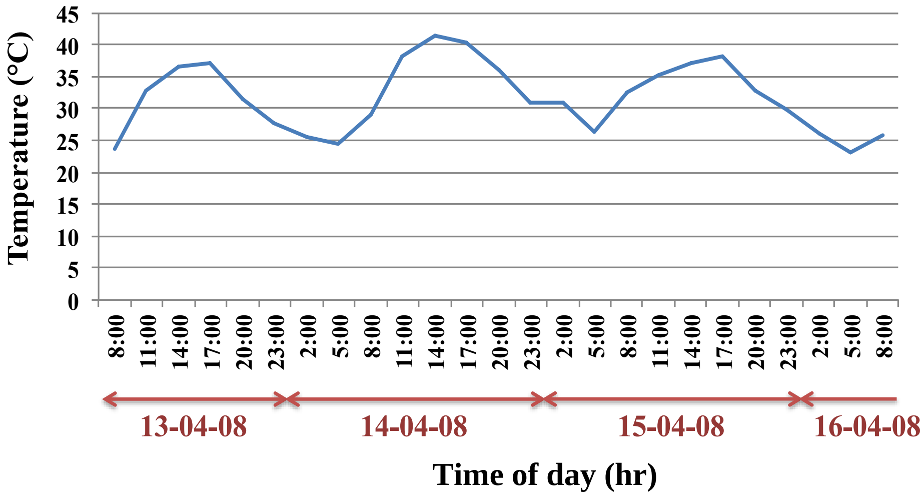

Fig. S3 Clustering dendrogram of module eigenvalues for *A. thaliana* and *A. hierochuntica* transcriptome profiles under heat stress conditions. Dendrograms were generated using the WGCNA package with module size minimum set at 50 genes. Dendrograms are presented after merging of modules with a cut off of 30% dissimilarity (70% similarity). These modules were assigned standard color-based names by WGCNA. Identical module names between *A. thaliana* and *A. hierochuntica* do not indicate similarity in function, expression profile or shared genes.

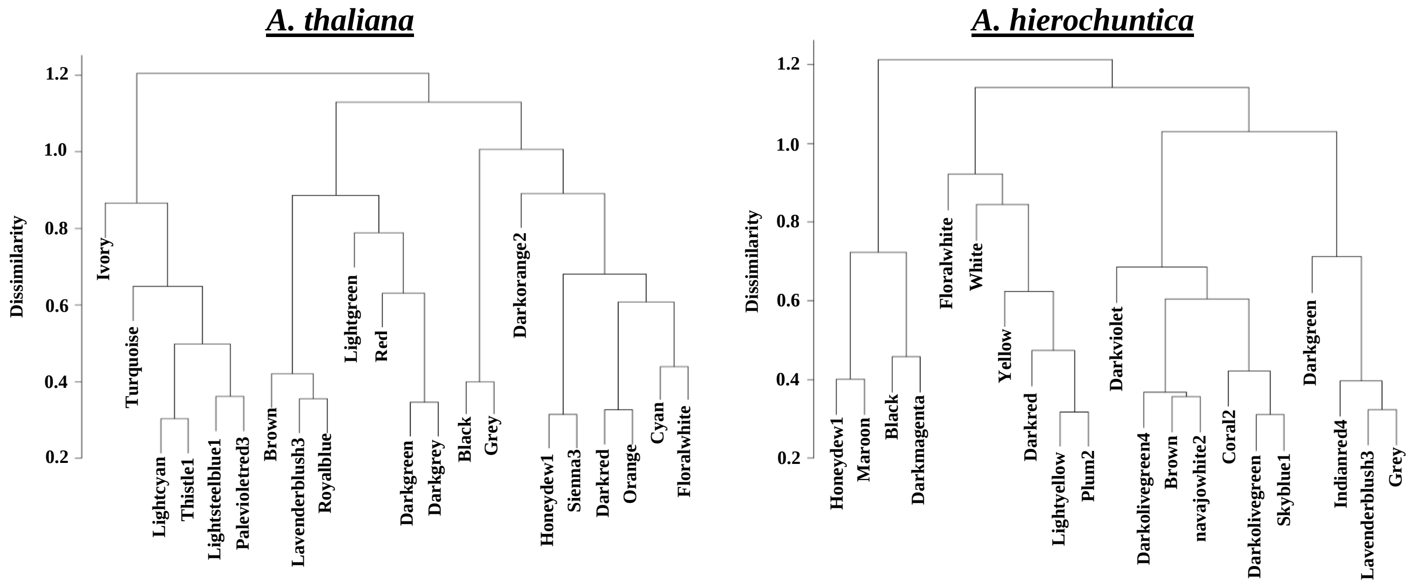

Fig. S4 Validation of 'between species' RNA-seq analysis. (a) Top 10 expressed transcripts as a percentage of all expressed transcripts. Data are mean ± SD (*n* = 3). (b) Comparison of basal expression of control samples using DeSeq2 rlog normalization or the between species Scale-Based Normalization (SCBN) method (Zhou *et al.*, 2019). Numbers next to boxes are median values. Letters above the circles indicate significant differences at $p < 0.05$ (Wilcoxon rank sum test) (c) Relative QPCR expression of selected *A. thaliana* and *A. hierochuntica* genes. Gene expression was determined according to the $2^{-\Delta\Delta C}T$ method (Livak & Schmittgen, 2001) using *eIF4A1* from each species as a reference gene. Expression was normalized to the expression level in the control morning sample, which was assigned a value of 1. Data are mean ± SD (*n* = 3 to 4) and are representative of two independent experiments. (d) Comparison of the basal (control) expression levels of *CPR5* estimated by RNA-seq or absolute QPCR quantification of transcript copy number. Absolute quantification was performed using a fivefold serial dilution of gel-purified *CPR5* and *eIF4A1* (reference gene) QPCR products to create a standard curve. CM, control morning; CN, control afternoon; HW1M, heat wave 1 morning; HW1N, heat wave 1 afternoon; HW2N, heat wave 2 afternoon; HW3N, heat wave 3 afternoon; R1N, day 1 recovery from heat stress afternoon; CR2N, control plants parallel to the R2N time point afternoon; R2N, day 2 recovery from heat stress afternoon. Blue shading, control conditions; Pink shading, heat conditions. *A.t*, *Arabidopsis thaliana*; *A.h*, *Anastatica hierochuntica*; TPM, transcripts per kilobase million.

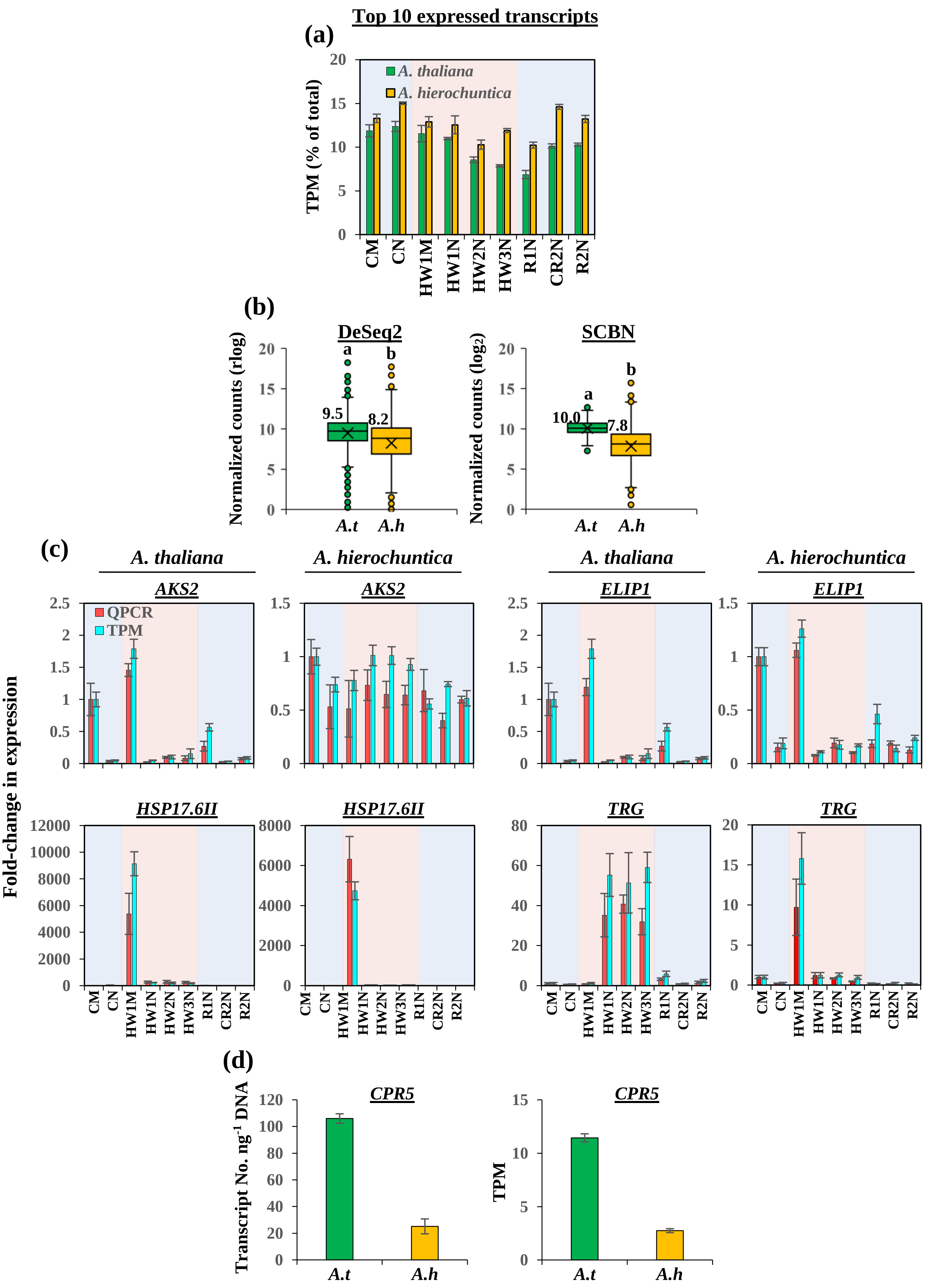

Top 10 expressed transcripts
(a)
TPM (% of total)
A. thaliana
A. hierochuntica
CM CN HW1M HW1N HW2N HW3N R1N CR2N R2N
(b)
DeSeq2
SCBN
Normalized counts (rlog)
Normalized counts (log2)
a
b
9.5
8.2
10.0
7.8
A.t
A.h
(c)
A. thaliana
A. hierochuntica
AKS2
ELIP1
HSP17.6II
TRG
QPCR
TPM
Fold-change in expression
(d)
CPR5
Transcript No. ng-1 DNA
TPM

Validation of 'between species' RNA-seq analyses

The above comparisons of gene expression between the two species utilized DeSeq2 (Love *et al*., 2014) as a normalized measure of gene expression and to identify differentially expressed genes. DeSeq2 normalizes read counts for different sequencing depths between samples. However, when dealing with two different species, several other factors can affect direct comparison of expression levels between orthologs including whether a few highly expressed genes constitute a large proportion of the sequenced transcripts, as well as differences in gene numbers and orthologous transcript length (Zhou *et al*., 2019; Zhao *et al*., 2020). Therefore, we performed several further analyses to validate our results. Figure S4a shows that there was no significant difference between the species in the proportion of the top 10 most highly expressed genes out of the total transcripts sequenced across all treatments (*A. thaliana*, ~7% to 12%, and *A. hierochuntica*, ~10% to 15% of the total sequenced transcripts). We further re-normalized our raw read count data (normalized for transcript length) using a new between-species method that applies Scale-Based Normalization (SCBN) to the most conserved orthologs, thereby obtaining a scaling factor that minimizes the false discovery rate of differentially expressed genes (Zhou *et al*., 2019). Applying SCBN to the 109 most conserved orthologs between *A. thaliana* and *A. hierochuntica* (Dataset S12) and using the scaling factor to correct normalized gene counts, we obtained similar comparative basal expression results as observed with DeSeq2 (Fig. S4b). Finally, QPCR analysis confirmed the RNA-seq fold-change gene expression patterns of four selected *A. thaliana* and *A. hierochuntica* genes (Fig. S4c). These genes included *AKS2,* a gene found to be positively selected in the 'all extremophyte species' run (Table 1), two genes involved in abiotic stress responses (*ELIP1* and *HSP17.6II*; Sun

et al., 2001; Rizza *et al*., 2011), and an *A. thaliana*-specific and an *A. hierochuntica*-specific Taxonomically Restricted Gene (*A. hierochuntica* ID: TRINITY_DN7044_c0_g2_i1; AGI: At2g07719; Methods S1). Additionally, we selected an ethylene signaling gene *CPR5* (Wang *et al*., 2017) that exhibited lower basal expression in *A. hierochuntica* than in *A. thaliana* in the RNA-seq analysis and confirmed this result via absolute QPCR (Fig. S4d).

References

Love MI, Huber W, Anders S. 2014. Moderated estimation of fold change and dispersion for RNA-seq data with DESeq2. *Genome Biology* **15:** 550.

Rizza A, Boccaccini A, Lopez-Vidriero I, Costantino P, Vittorioso P. 2011. Inactivation of the *ELIP1* and *ELIP2* genes affects *Arabidopsis* seed germination. *New Phytologist* **190:** 896-905.

Sun W, Bernard C, van de Cotte B, Van Montagu M, Verbruggen N. 2001. *At-HSP17.6A*, encoding a small heat-shock protein in *Arabidopsis*, can enhance osmotolerance upon overexpression. *The Plant Journal* **27:** 407-415

Wang F, Wang L, Qiao L, Chen J, Pappa MB, Pei H, Zhang T, Chang C, Dong C-H. 2017. *Arabidopsis* CPR5 regulates ethylene signaling via molecular association with the ETR1 receptor. Journal of Integrative Plant Biology **59:** 810-824.

Zhao S, Ye Z, Stanton R. 2020. Misuse of RPKM or TPM normalization when comparing across samples and sequencing protocols. *RNA* **26:** 903-909.

Zhou Y, Zhu J, Tong T, Wang J, Lin B and Zhang J. 2019. A statistical normalization method and differential expression analysis for RNA-seq data between different species. BMC Bioinformatics **20:** 163.

Fig. S5 Basal (control) expression of 15 orthologous *A. thaliana* and *A. hierochuntica* housekeeping genes. (a) RNA-seq-based expression (see Methods S1). (b) Ratio of *A. thaliana*:*A. hierochuntica* expression.

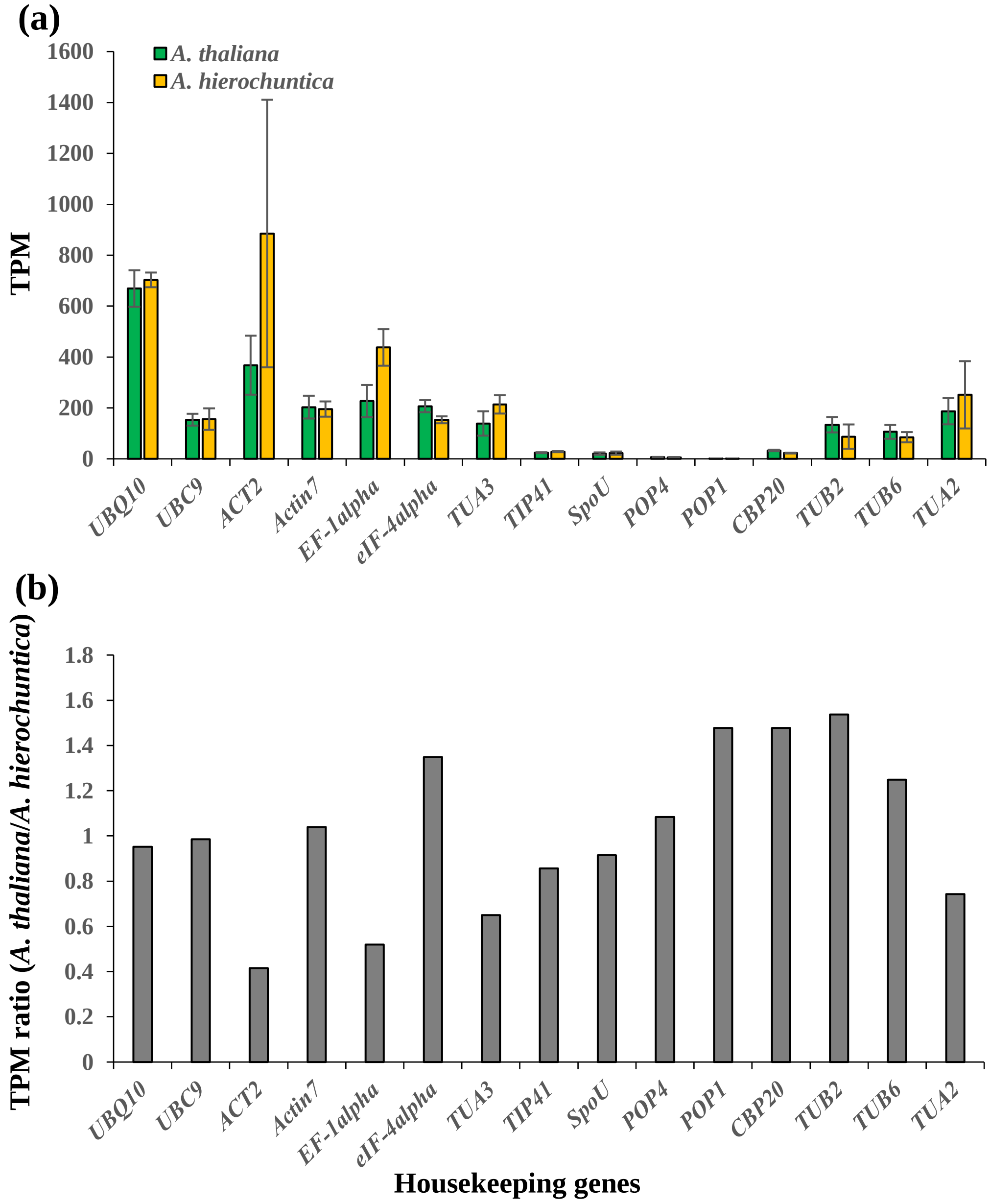

Fig. S6 Number of protein-coding transcripts and comparative ortholog group composition for species used to detect positively selected genes. Data were generated using the OrthoFinder software (Emms & Kelly, 2019).

Reference

Emms DM, Kelly S. 2019. OrthoFinder: phylogenetic orthology inference for comparative genomics. *Genome Biology* **20:** 238.

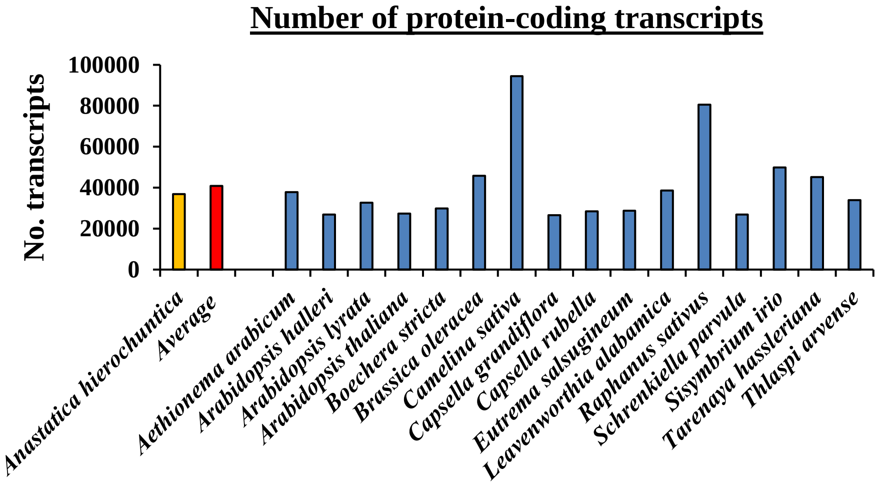

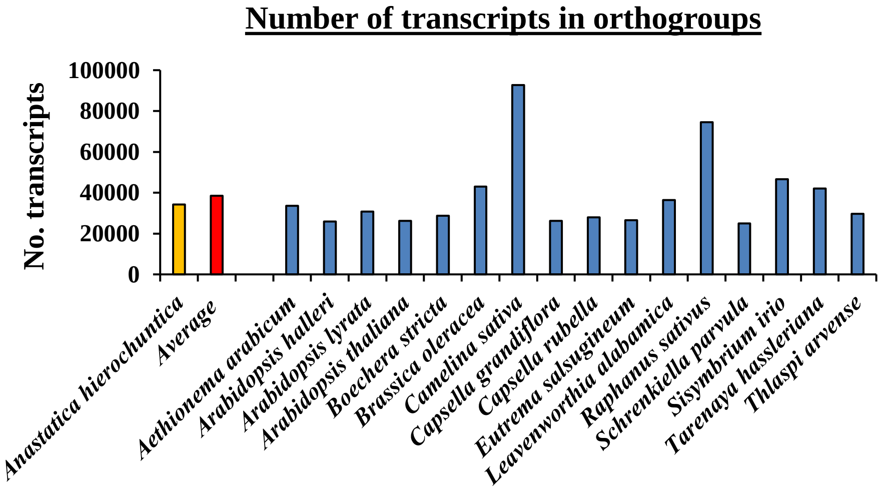

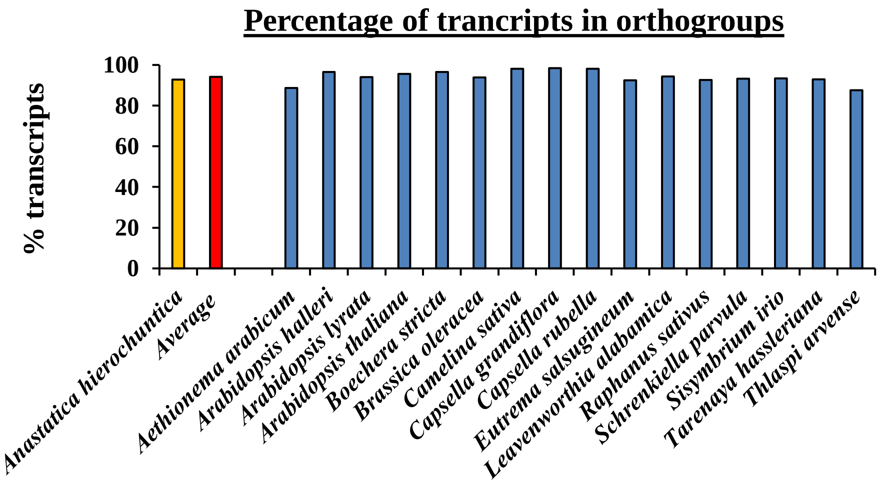

New Phytologist

Fig. S7 GO-term enrichment analysis of positively selected genes. Significantly (q-value < 0.05) enriched GO terms (biological process and molecular function) were compared among the five positive selection analyses and are represented in a heatmap. GO terms were assigned based on the *A. thaliana* ortholog sequence, or closest paralog. The *A. thaliana* genome served as the background for the enrichment analysis which was performed using the AgriGO online server (http://bioinfo.cau.edu.cn/agriGO/analysis.php). The red color intensity corresponds to the number of positively selected genes assigned with that GO term (the numbers are indicated within the cells). Cells with a white color correspond to GO terms that were not significantly enriched. For visualization and interpretation purposes, GO terms with > 2000 genes in the *A. thaliana* genome were excluded. Additionally, GO terms were clustered together if they share >50% of their gene set, and the GO term with the lowest q-value per cluster was included in the heatmap (Full enriched GO term lists can be found in Datasets S21-S24).

Biological Process

11	9	8	5	Anatomical structure development (GO:0048856)
21	19	16	14	Biological regulation (GO:0065007)
29	29	21	22	Biosynthetic process (GO:0009058)
29	28	21	21	Cellular biosynthetic process (GO:0044249)
15	24	17	14	Cellular macromolecule biosynthetic process (GO:0034645)
24	34	24	24	Cellular macromolecule metabolic process (GO:0044260)
8	12	10	11	Cellular protein metabolic process (GO:0044267)
11	13	9	8	Developmental process (GO:0032502)
16	24	19	14	Gene expression (GO:0010467)
15	24	17	14	Macromolecule biosynthetic process (GO:0009059)
25	36	26	28	Macromolecule metabolic process (GO:0043170)
10	13	8	8	Multicellular organismal development (GO:0007275)
10	13	8	8	Multicellular organismal process (GO:0032501)
24	28	23	19	Nitrogen compound metabolic process (GO:0006807)
19	23	19	15	Nucleobase, nucleoside, nucleotide and nucleic acid metabolic process (GO:0006139)
9	12	12	15	Protein metabolic process (GO:0019538)
18	19	12	12	Regulation of biological process (GO:0050789)
11	14	9	8	Regulation of biosynthetic process (GO:0009889)
11	14	9	8	Regulation of cellular biosynthetic process (GO:0031326)
13	14	9	10	Regulation of cellular metabolic process (GO:0031323)
18	18	11	12	Regulation of cellular process (GO:0050794)
11	15	9	8	Regulation of gene expression (GO:0010468)
11	14	9	8	Regulation of macromolecule biosynthetic process (GO:0010556)
12	15	9	8	Regulation of macromolecule metabolic process (GO:0060255)
13	15	9	10	Regulation of metabolic process (GO:0019222)
11	13	9	8	Regulation of nitrogen compound metabolic process (GO:0051171)
11	13	9	8	Regulation of nucleobase, nucleoside, nucleotide and nucleic acid metabolic process (GO:0019219)
12	14	9	8	Regulation of primary metabolic process (GO:0080090)
11	13	9	8	Regulation of transcription (GO:0045449)
8	14	8	6	Response to chemical stimulus (GO:0042221)
6	10	5	5	Response to organic substance (GO:0010033)
14	20	14	11	Response to stimulus (GO:0050896)
9	11	12	6	RNA metabolic process (GO:0016070)
11	16	11	9	Transcription (GO:0006350)
7	8	6		Post–embryonic development (GO:0009791)
9	10	8		Response to stress (GO:0006950)
5				Anatomical structure morphogenesis (GO:0009653)
5				Monocarboxylic acid metabolic process (GO:0032787)
	5			Macromolecule modification (GO:0043412)
	5			Protein modification process (GO:0006464)
		5		Lipid biosynthetic process (GO:0008610)
7	5			Cellular component biogenesis (GO:0044085)
8	7			Establishment of localization (GO:0051234)
8	7			Transport (GO:0006810)
6			6	Cellular nitrogen compound metabolic process (GO:0034641)
7			6	Heterocycle metabolic process (GO:0046483)
8			7	Localization (GO:0051179)
	5	5		Translation (GO:0006412)
				Carboxylic acid biosynthetic process (GO:0046394)
				Cell growth (GO:0016049)
				Cellular component morphogenesis (GO:0032989)
				Cellular developmental process (GO:0048869)
				Cellular response to stimulus (GO:0051716)
				Cellular response to stress (GO:0033554)
				Embryonic development (GO:0009790)
				Embryonic development ending in seed dormancy (GO:0009793)
				Fruit development (GO:0010154)
				Growth (GO:0040007)
				Negative regulation of biological process (GO:0048519)
				Organic acid biosynthetic process (GO:0016053)
				Regulation of anatomical structure size (GO:0090066)
				Regulation of cell size (GO:0008361)
				Regulation of cellular component size (GO:0032535)
				RNA biosynthetic process (GO:0032774)
				Seed development (GO:0048316)
				Transcription, DNA–dependent (GO:0006351)
5				Cellular nitrogen compound biosynthetic process (GO:0044271)
5				Cofactor biosynthetic process (GO:0051188)
5				Cofactor metabolic process (GO:0051186)
5				Intracellular transport (GO:0046907)
	5			Post–translational protein modification (GO:0043687)
	7			Response to abiotic stimulus (GO:0009628)
	5			Response to abscisic acid stimulus (GO:0009737)

Molecular Function

	A. hierochuntica	*E. salsugineum*	*S. parvula*	*A. thaliana*
Cation binding (GO:0043169)	10	8	10	6
Ion binding (GO:0043167)	10	8	10	6
Metal ion binding (GO:0046872)	10	8	9	6
Transcription factor activity (GO:0003700)	11	14	6	11
Transferase activity (GO:0016740)	14	15	8	12
Transition metal ion binding (GO:0046914)	6	7	8	6
Protein binding (GO:0005515)	13	14		12
Transferase activity, transferring phosphorus–containing groups (GO:0016772)	7	8		6
DNA binding (GO:0003677)	16	19		
Zinc ion binding (GO:0008270)			5	5
Oxidoreductase activity (GO:0016491)	11	10	8	
Nucleic acid binding (GO:0003676)		27		
Transcription regulator activity (GO:0030528)		16		
Acid–amino acid ligase activity (GO:0016881)				
Kinase activity (GO:0016301)	6			
Lyase activity (GO:0016829)	6			
Transporter activity (GO:0005215)	8			
Structural molecule activity (GO:0005198)		7		

Methods S1 Additional information regarding methods used in this study.

Plant material and growth conditions

F_4 generation *A. hierochuntica* seeds descended from a single seed from plants originally collected in the Negev Desert (Nahal Hayun, 30.191424N and 35.009926E), Israel, were used in this study.

Seeds for plants used to extract RNA for sequencing of the *de novo* reference transcriptome were germinated and grown on nutrient agar plates for 5 d in the growth room (16 h light (150 µmol photons m^{-2} s^{-1})/8 h dark; 22 °C), as described in Eshel *et al*. (2017). Plant material was prepared for sequencing on two platforms: (i) For Illumina sequencing, plate-grown seedlings were harvested and snap-frozen in liquid nitrogen; (ii) For Roche 454 sequencing, plate-grown 5 day-old seedlings were transferred to pots containing autoclaved *A. thaliana* soil growth medium (Weizmann Institute of Science), irrigated to field capacity with 1 g l^{-1} 20-20-20 NPK + micronutrients solution (Haifa Chemicals), and kept in the growth room until plants developed four true fully-expanded leaves. These plants were then treated with the following conditions: (a) Control (field-capacity, 22 °C); (b) Drought stress (25% field capacity for 1 week); (c) Salt shock (200 mM NaCl in the fertilizer solution), harvested after 1, 3 and 6 h; (d) Heat shock (45 °C), harvested after 0.5, 1, and 2 h. Roots, shoots and flowers (where available) from these soil-grown plants, were harvested separately and snap-frozen in liquid nitrogen. In addition to these soil-grown samples, mature seeds, from the same F_4 generation seed stock, were imbibed in H_2O for 8.5 h, and then snap-frozen in liquid nitrogen.

For RNA-seq heat stress experiments, *A. thaliana* and *A. hierochuntica* were germinated and grown on nutrient agar plates according to Eshel *et al*. (2017). Seedlings were grown on

plates until cotyledons were fully expanded before transfer to 7 cm x 7 cm x 8 cm pots containing *Arabidopsis* nitrogen-less soil (Weizmann Institute of Science; 70% fine peat [1–10mm], 30% perlite 4) irrigated to field capacity with a custom-made fertilizer solution (5 mM KNO_3, 2 mM $MgSO_4$, 1 mM $CaCl_2$ x $2H_2O$, 10 mM KH_2PO_4 [pH 6.0, adjusted with KOH] plus MS micronutrients (Murashige & Skoog, 1962). Flats containing pots were placed in the growth room under the same conditions as for the plate experiments. Flats were covered with plastic domes for 1 to 2 d, which were then gradually removed to allow seedlings to harden. Each day, pots were shuffled so that all plants received equal illumination and to remove shelf position effects. Plants were irrigated alternatively every 3 d with either fertilizer solution or water in order to maintain constant nutrient concentrations. After 6 d in the growth room, uniform plants were transferred to two growth chambers (KBWF 720, BINDER GmbH, Tuttlingen, Germany) (16 h light/8 h dark; 23 °C; 60% relative humidity) for heat treatments. The light/dark transitions at the beginning and end of the day comprised 0.5 h at 100 µmol photons m^{-2} s^{-1} and 0.5 h at 150 µmol photons m^{-2} s^{-1} to mimic sunrise and sunset. Light intensity for the remaining 14 h was 250 µmol m^{-2} s^{-1}. Plants were allowed to acclimate for 4 d and were moved randomly every day between the chambers, to avoid chamber effects. At day 10 after transfer to soil, (*A. hierochuntica* plants had two true leaves and *A. thaliana* had six true leaves), heat treatment was initiated in one chamber, keeping the other chamber as the control (23 °C). The heat treatment included 3 d at 40/25 °C, day/night temperatures, followed by two days of recovery at control conditions (Fig. 2A). Similar to the 1 h light transition, the temperature was also gradually increased/decreased for 1 h, between the light/dark states, to reach the appropriate temperatures. Plants from both chambers were harvested at eight time points (Fig. 2A), either

in the morning (1.5 h after the onset of the light/heat period) or at midday (7 h after the onset of the light/heat period). For each condition, three biological replicates comprising 6 pooled plants per replicate (27 samples per species) were used for downstream analyses.

Reference transcriptome sequencing, assembly and annotation

To generate a high-quality *A. hierochuntica* reference transcriptome that maximizes coverage of genes contained in the genome, we sequenced RNA pooled from multiple plant organs (root, shoot, flower, seeds), at different developmental stages (early seedling stage, and mature plants before and after anthesis), and under control and stress conditions (heat, drought and salinity).

For Illumina sequencing, a cDNA library was prepared using the TruSeq Stranded mRNA Sample Preparation kit (Illumina, San Diego, CA), according to the manufacturer's instructions. The library was then sequenced on Illumina Genome Analyser IIx (GAIIx), where 36,666,369 single-end, quality filtered, unaligned 76 nucleotide (nt) long raw reads, were generated with CASAVA 1.8 software. Reads containing adapter sequences were discarded, resulting in 36,369,571 filtered reads.

For Roche '454' sequencing, a normalized cDNA library was constructed using the Evrogen SMART technology cDNA synthesis service (Evrogen, Moscow, Russia) to reduce abundant RNAs such as Rubisco, and therefore enable detection of rare RNAs. The library was sequenced with a full picotiter plate on a 454 GS-FLX sequencer with Titanium reagents (Roche Applied Science, Indianapolis, IN, USA), yielding 862,788 raw reads (average read length of 315 nt). Read quality was monitored using FastQC (Babraham Institute,

http://www.bioinformatics.babraham.ac.uk/projects/fastqc/). Raw reads were trimmed and filtered using the CLC Genomic Workbench (CLC bio, Inc.) with the following parameters: (i) Quality limit = 0.05; (ii) Ambiguous limit = 2; (iii) Minimum number of nucleotides in reads = 30; (iv) Adapter removal. To remove potential contaminant sequences, human, mouse, mosquito, bacteria, archaea and viruses sequence databases were screened using DeconSeq (Schmieder and Edwards, 2011) with alignment coverage length and identity thresholds of 95% and 94%, respectively. The potential contaminants were further BLASTN against the RefSeq_rna, and the TAIR10 databases, and searched using MEGABLAST against the nr databases, where reads with significant homology (e-value < $1e^{-5}$) to a plant sequence were retained. To account for potential inherent homopolymer insertion/deletion sequencing errors in the '454' reads, Illumina reads were used for error correction using the Blue error-correction algorithm (Greenfield *et al*., 2014). Altogether 724,653 high quality '454' reads were obtained. All Illumina and 454 reads were deposited in the SRA database under NCBI BioProject PRJNA731383.

The reference transcriptome was assembled using a hybrid assembly approach that utilized both Illumina and 454 reads. Briefly, the Illumina reads were first assembled using the Trinity software package (Grabherr *et al*., 2011). The resulting Trinity contigs were then shred into 700 bp fragments ('pseudo-reads'), with a 200 bp overlap between adjacent fragments, using the fasta2frag.tcl script, from the MIRA4 package (Chevreux *et al*., 1999; Chevreux *et al*., 2004). Both the shredded contigs and the 454 reads were assembled together using the Newbler assembler v.2.6 (Margulies *et al*., 2005) to generate the *A. hierochuntica* reference transcriptome comprising 36,871 assembled high-confidence transcripts (Figure S1).

Transcriptome completeness was assessed using the Benchmarking Universal Single-

Copy Orthologs (BUSCO) tool (Simao, *et al*., 2015). TransDecoder (Haas *et al*., 2013) was used for predicting coding sequences in the Newbler-assembled transcripts. In order to assess the utility of the reference transcriptome for gene expression quantification, the Illumina reads were mapped to the reference *A. hierochuntica* transcriptome using the Bowtie read aligner program (Langmead *et al*., 2009), with the seed length set to 40 nt, and the maximum mismatches allowed within the seed length set to one.

For functional annotation, transcripts were searched for homolog sequences in other species using BLAST (e-value ≤ $1e^{-5}$ cutoff) against the *A. thaliana* (TAIR10) coding sequences (CDS) database, a custom-made *Brassicaceae* CDS database (for included species see Dataset S14), and the curated, non-redundant RefSeq_protein and RefSeq_rna databases. Transcripts were also BLAST against the ncRNA databases, CANTATAdb (Szcześniak *et al*., 2015) and NONCODE2016 (Zhao *et al*., 2015). For these transcripts, ncRNA structures were also predicted using the online tool RNAcon (Panwar *et al*., 2014; http://crdd.osdd.net/raghava/rnacon/). Transcripts were also mapped to the Kyoto Encyclopedia of Genes and Genomes (KEGG) pathway database using the KAAS server (Moriya *et al*., 2007), with the bi-directional best-hit method against plant species (Zhang & Leong, 2010). The PlantTFcat server (Dai *et al*., 2013) was used for identifying regulatory genes (transcription factors (TFs), transcriptional regulators (TRs) and chromatin regulators (CRs). Transcripts were also searched for InterPro protein signatures using the InterProScan function (with the following applications: BlastProDom, FPrintScan, HMMPIR, HMMPfam, HMMSmart, HMMTigr, ProfileScan, HAMAP, PartterScan, SuperFamily, SignalPHMM, TMHMM, HMMPanther, Gene3D, Phobius and Coils) within the Blast2GO program (Conesa *et al*., 2005).

Functional annotation procedure	Total annotated transcripts
Coding sequence prediction	28,729 (78%)
InterPro signatures (InterProScan)	27,511 (75%)
KEGG pathways (KAAS)	9,010 (24%)
Plant regulatory gene families (PlantTFcat)	4,043 (11%)
Best hit to *Arabidopsis thaliana* TAIR10 (BLASTN)	25,386 (69%)
Best hit to *Brassicaceae* species (BLASTN)	29,775 (81%)
Best hit to RefSeq_rna (BLASTN)	33,263 (90%)
Best hit to RefSeq_protein (BLASTX)	33,232 (90%)
Total annotated	35,472 (96%)

Identification of *A. thaliana* and *A. hierochuntica* taxonomically restricted genes (TRGs)

A. thaliana and *A. hierochuntica* transcriptomes were translated into ORFs and compiled into protein databases for the two species using TransDecoder (Haas *et al*., 2013). Reciprocal BLASTp (ver. 2.10.1) was performed against the protein databases of 25 plant species with the following parameters: E-value ≤ $1e^{-5}$, Max. sequence target = 1. After compiling the transcripts that were not annotated, we performed BLASTn (ver 2.10.1) using the same parameters as above against the *A. thaliana* genome to further filter out transcripts which might have a putative function.

RNA-seq of heat responsive *A. thaliana* and *A. hierochuntica* transcriptomes

RNA-seq libraries were prepared with the Illumina 'TruSeq Stranded mRNA Sample Prep Kit (Illumina), multiplexed and pooled for each species separately (27 samples per species) and sequenced across 9 and 11 lanes for *A. thaliana* and *A. hierochuntica*, respectively. Sequencing

was performed using an Illumina HiSeq2500 sequencer to generate 100nt single-end reads. In addition, a pooled sample of equal amounts of RNA from all *A. hierochuntica* samples, was prepared and sequenced generating 160nt paired-end reads. Fastq files were generated and demultiplexed with the bcl2fastq v2.17.1.14 Conversion Software (Illumina), while the FastQC program was used to monitor read quality. RNA-seq reads were deposited in the SRA database under NCBI BioProject PRJNA731383.

To estimate transcript abundance, 27 RNA-Seq *A. hierochuntica* fastq files were mapped to the reference transcriptome, while 27 RNA-Seq *A. thaliana* fastq files were mapped to the TAIR10 33,602 cDNA representative sequences. This was achieved using the Trinity align_and_estimate_abundance.pl script, which applies the RSEM program (Li & Dewey, 2011) with the Bowtie aligner. RSEM calculates the transcripts per kilobase million (TPM) normalized gene expression estimations, TPM values were used for individual gene expression graphs, while $\log_{10}$ transformed TPM values were used for the PCA analysis.

For statistical differential expression tests, count data of uniquely mapped reads were calculated per transcript from the Bowtie output using a custom python script, and were further analyzed using the R package DESeq2 (Love *et al*., 2014). In order to test for differences between species, a subset of 17,962 Bi-directional Best BLASTN hits (orthologs) between the two species, was used. DESeq2 normalizes the count data of each library by a size factor to account for different sequencing depths between the libraries. However, when comparing the expression levels between different species, it is essential to also normalize the read counts by the transcript length. Therefore, instead of inputting DESeq2 with the read counts per transcript, we first divided these values by the transcript length (in kilobases) and rounded the

values to input DESeq2 with discrete numbers that fit a Poisson distribution.

One inherent problem in analyzing expression data (or any other type of multidimensional data) is that the variance increases with the mean. Therefore, the most highly expressed genes will dominate the differential expression analysis. One common solution is to use logarithmic transformation but this approach is prone to dominance of the lowest expressed genes, which will show the strongest differences between samples (see DESeq2 manual, http://www.bioconductor.org/help/workflows/rnaseqGene/#time-courseexperiments). Therefore, DESeq2 uses the regularized-logarithm transformation (rlog) to stabilize the variance in count data across the mean, thereby improving the dispersion estimation for both high and low expressed genes, and reducing the false discovery rate in calling differential expression (Love *et al.*, 2014).

For analysis modes of expression and expression of genes associated with specific functions (Figs. 3A, 4 and 5B), genes were assigned GO terms for their respective categories using the BiNGO app of the Cytoscape software (Maere *et al.*, 2005). The GO terms used for cell cycle were: ‘cell cycle’ (GO:0007049), ‘cytokinesis’ (GO:0000910), ‘regulation of cellular division’ (GO:0051302), ‘cell division’ (GO:0051301); The GO terms used for photosynthesis were: ‘photosynthesis’ (GO:0015979), ‘photorespiration’ (GO:0009853), ‘electron transport chain’, (GO:0022900) ‘light reaction’ (GO:0019684), ‘oxidation reduction’ (GO:0055114); The GO terms used for abiotic stress were: ‘response to water deprivation’ (GO:0019684), ‘resp. to heat’ (GO:0009408), ‘resp. to temperature stimulus’ (GO:0009266), ‘resp. to reactive oxygen species’ (GO:0000302), ‘resp. to hydrogen peroxide’ (GO:0042542), ‘resp. to high light intensity’ (GO:0009644), ‘resp. to oxidative stress’ (GO:0006979), ‘resp. to radiation’ (GO:0009314), ‘resp.

to. abscisic acid stimulus' (GO:0009737), 'resp. to osmotic stress' (GO:0006970), and 'regulation of response to stress (GO:0080134)'.

For functional clustering of GO-terms in Fig. 5C, the GOMCL algorithm (Wang *et al*., 2020) was used to reduce redundancy of the Gene Ontology. GO terms that share the majority (>50%) of genes among them were clustered with an inflation value of 3. Enriched GO terms with more than 1,000 genes in the *A. thaliana* genome were excluded.

Validation of RNA-seq data

Scale-based normalization (SCBN)

To validate the differential gene expression analysis performed with DeSeq2, we re-normalized our RNA-seq read count data (normalized to transcript length) using the Scale-Based Normalization (SCBN) method that aids in removing systematic variation between different species (Zhou *et al*. 2019), For the first step - pinpointing highly conserved genes - we utilized the 17,962 *A. thaliana* and *A. hierochuntica* orthologous genes identified using the Agalma phylogenomics pipeline (see below 'Phylogenomics and positive selection analysis'). Applying BLASTn (ver, 2.10.1) software revealed 109 highly conserved orthologs with an E-value ≤ $1e^{-100}$, query coverage of ≥ 98%, and identical matches of ≥ 99%. The SCBN R package (http://www.bioconductor.org/packages/devel/bioc/html/SCBN.html) was then used on the 109 highly conserved genes to obtain a scaling factor of 0.9223461, which it then applied to the 17,962 orthologs to call 12,808 common differentially expressed genes ($p \leq 1e^{-05}$) between the two species. To generate an approximate corrected gene count, the scaling factor was applied to each individual gene and the corrected average and median basal (control) expression is

depicted in Fig. 9B.

Real-time QPCR analysis

Total RNA was extracted from whole shoots with TRIzol (38% Phenol (w/v), 0.8 M guanidine thiocyanate, 0.4 M ammonium thiocyanate, 0.1 M sodium acetate pH 5, 5% glycerol (v/v)) according to Rio *et al*. (2010). To remove residual genomic DNA, 7 [illegible]g of total RNA was treated with RNase-free PerfeCta DNase I (Quanta Biosciences, Inc., Gaithersburg, MD, USA) according to the manufacturer's instructions, and cDNA was synthesized from 1 µg of total RNA using the qScript^{TM} cDNA Synthesis Kit (Quanta Biosciences). For amplification of PCR products, primers were designed using the NCBI Primer-BLAST tool (Ye *et al*., 2012), and analyzed for any secondary structure with the IDT OligoAnalyzer™ Tool (Dataset S13). QPCR was performed with the ABI PRISM 7500 Sequence Detection System (Applied Biosystems). Each reaction contained 5 µl Applied Biosystems™ Power SYBR® Green PCR Master Mix (Thermo Fisher Scientific Inc., Waltham, MA, USA), 40 ng cDNA, and 300 nM of each gene-specific primer. The QPCR amplification protocol was: 95 °C for 60 s, 40 cycles of 95 °C for 5 s (denaturation) and 60 °C for 30 s (annealing/extension). Data were analyzed using the SDS 2.3 software (Applied Biosystems). To check the specificity of annealing of the primers, dissociation kinetics was performed at the end of each PCR run. All reactions were performed in triplicates. Relative quantification of target genes was calculated using the $2^{-\Delta\Delta C}$T method (Livak & Schmittgen, 2001), using *A. thaliana eIF4A1* and the *A. hierochuntica eIF4A1* ortholog as internal references. To ensure the validity of the $2^{-\Delta\Delta C}$T method, standard curves of 2-fold serial dilutions of cDNA were created and amplification of efficiencies of target and reference gene were shown to be

approximately equal. For absolute quantification of basal expression, QPCR products were gel-purified (Gel/PCR DNA Fragments Extraction Kit [Geneaid Biotech Ltd, New Tapei City, Taiwan]), and quantified with a Nanodrop spectrophotometer. Fivefold serial dilutions of each PCR product were used to create a standard curve for determination of transcript copy number. As a loading control, the absolute transcript copy number of *eIF4A1*was also calculated and normalized to the highest *eIF4A1*level, which was assigned a value of 1. The target gene transcript copy number was then adjusted for loading differences by dividing by the normalized *eIF4A1* level.

Clustering by Weighted Gene Correlation Network Analysis (WGCNA) and GO enrichment

WGCNA was performed according to Langfelder & Horvath (2008) using R version 3.6.1 in RStudio (version 1.2.1335 (RStudio, Inc.)). WGCNA was set to using a weighted network analysis with Pearson correlations. The soft thresholding power (argument 'power' in function 'Adjacency') was set to 24 for *A. thaliana* and 30 for *A. hierochuntica*. Minimum module size (argument 'minClusterSize' in function 'dynamicMods') was set to 50 for both species, and module merging height cut (argument 'cutHeight' in function 'mergeCloseModules') was set to 0.30 for both species. Module expression profile heatmaps were produced from data created by WGCNA using the R package pheatmap (Kolde, 2012). A custom script was written to scale the expression data of each module's constituent genes relative to the other genes in the module so that expression pattern rather than expression level was visualized.

Gene Ontology (GO) enrichment analysis of the heat shock modules was performed using the Cytoscape (version 3.7.1) software app BiNGO (Shannon *et al*., 2003; Maere *et al*.,

2005). For *A. thaliana*, a significance level of 0.05 was used, with an overrepresentation of ≥ 1.5. For *A. hierochuntica*, the respective *A. thaliana* orthologs were used. To correct for any high GO-term enrichment bias in *A. hierochuntica*, orthologs of the whole annotated *A. hierochuntica* transcriptome were tested for GO enrichment. The highest level of GO term enrichment was 1.38. Since for *A. thaliana* GO terms are usually only considered enriched at ≥ 1.5-fold ($q \leq 0.05$), we corrected for any bias in the *A. hierochuntica* transcriptome by using a cutoff of ≥ 2.0 (1.5 * 1.38 ≈ 2.0)) with $q \leq 0.05$.

Phylogenomics and positive selection analysis

To identify positively selected genes, which are unique to *A. hierochuntica* or common to extremophyte Brassicaceae species, we used coding sequences of the *A. hierochuntica* reference transcriptome and 16 sequenced Brassicaceae species (Dataset S14) into the automated Agalma phylogenomics pipeline (Dunn *et al*., 2013).

The Agalma pipeline identified orthologous genes among these species by: (i) Identifying homologous genes among all input sequences from all the species using an all-by-all TBLASTX search followed by a Markov Clustering Algorithm (MCL) tool (Enright *et al*., 2002); (ii) For each homolog group, a peptide multiple sequence alignment (MSA) is produced using MAFFT with the E-INS-i algorithm (Katoh *et al*., 2005); (iii) The MSA is further used to build a maximum likelihood (ML) phylogenetic tree with RAxML v8.2.3 (Stamatakis, 2014). (iv) The homolog group tree is further pruned into maximally inclusive subtrees to define ortholog groups. MSAs of 13,806 ortholog groups with a sequence representation in at least 4 taxa were concatenated into a supermatrix for ML species tree search, using RAxML (with the PROTGAMMAWAG model

of evolution, and 100 rapid bootstrap searches) under the WAG rate matrix (Whelan & Goldman, 2001), with gamma-distributed among-site rate variation.

To detect positive selection in the five extremophyte species, ortholog groups with sequence representation in at least 2 extremophytes, were selected to ensure sufficient statistical power (Anisimova *et al*., 2001). For each ortholog group, the peptide MSA was converted into the corresponding codon alignment using the pal2nal.pl program (Suyama *et al*., 2006), and the ML species tree was pruned using PHAST tree_doctor (Hubisz *et al*., 2011), to keep only sequence-represented taxa. Codon alignments together with pruned trees were further analyzed with the PAML v4.8, CODEML program (Yang, 1997; Yang, 2007), using the Branch-Site model. To test for positive selection, the tested branch(s) were labeled (foreground), and the log likelihood of two models (M1a and M2a), were calculated for each ortholog group. The difference between the two models is that in the M1a (null) model, the non-synonymous to synonymous rate ratio (dN/dS) is fixed to 1 (fix_omega = 1 and omega = 1), indicative of neutral selection, while in the M2a (alternative) model, the initial dN/dS ratio is set to 1, and is further estimated by the model (fix_omega = 0 and omega = 1). A Likelihood Ratio Test was performed (with X^2 distribution), to identify genes with log likelihood values significantly different between the two models, indicative of deviation from neutral selection. Ortholog groups with portion of sites in the foreground branches, that had an estimated dN/dS ratio greater than 1, were considered under positive selection. To account for multiplicity, a Benjamini–Yekutieli false discovery rate (FDR) correction (Benjamini & Yekutieli, 2001) was applied using the ‘qvalue’ R package, with a *q*-value < 0.05 cutoff for a gene to be considered as positively selected. Sites under positive selection were identified using the empirical Bayes

approach with a posterior probability $p > 0.95$.

The above procedure was repeated to identify positive selected genes in *A. hierochuntica*, and in the other extremophyte species. For each analysis, different branches on the tree were tested (labeled as foreground) compared with all other branches (background): (i) labeling the external branches of all five extremophyte species as the foreground (4,723 ortholog groups); (ii) labeling the *A. hierochuntica* external branch as the foreground (3,093 ortholog groups); (iii) labeling the *E. salsugineum* external branch as the foreground (4,457 ortholog groups); (iv) labeling the *S. parvula* external branch as the foreground (4,369 ortholog groups); and (v) labeling the *A. thaliana* external branch as the foreground (5,513 ortholog groups). *A. thaliana* was considered as a control/comparator species sensitive to abiotic stresses (Kazachkova *et al*., 2018). The Venn diagram comparing positive selected genes (Fig. 6B) was generated using an online tool: http://bioinformatics.psb.ugent.be/webtools/Venn/).

To further assess the functionality of the positively selected genes, Gene Ontology (GO) terms were assigned to each ortholog group based on *A. thaliana* GO annotation. In cases where an ortholog group did not contain an *A. thaliana* ortholog, the closest *A. thaliana* homolog (best BLASTP hit) was used. Significant positively selected genes were further tested for enriched GO terms (Fisher's exact test, with a q-value < 0.05 cutoff) using the online AgriGO tool (Du *et al*., 2010; http://bioinfo.cau.edu.cn/agriGO/analysis.php), where the *A. thaliana* genome served as the background. Enriched GO terms with more than 2,000 genes in the *A. thaliana* genome were excluded, as these are broad and less informative terms.

References

Anisimova M, Bielawski JP, Yang Z. 2001. Accuracy and power of the likelihood ratio test in detecting adaptive molecular evolution. *Molecular Biology & Evolution* **18:** 1585-1592.

Benjamini Y, Yekutieli D. 2001. The control of the false discovery rate in multiple testing under depencency. *Annals of Statistics* **29:** 1165-1188.

Chevreux B, Wetter T, Suhai S. 1999. Genome sequence assembly using trace signals and additional sequence information. *German Conference on Bioinformatics*. **99:** 45-56

Chevreux B, Pfisterer T, Drescher B, Driesel AJ, Müller WEG, Wetter T, Suhai S. 2004. Using the miraEST assembler for reliable and automated mRNA transcript assembly and SNP detection in sequenced ESTs. *Genome Research* **14:** 1147-1159.

Conesa A, Götz S, García-Gómez JM, Terol J, Talón M, Robles M. 2005. Blast2GO: a universal tool for annotation, visualization and analysis in functional genomics research. Bioinformatics **21:** 3674-3676.

Dai X, Sinharoy S, Udvardi M, Zhao PX. 2013. PlantTFcat: an online plant transcription factor and transcriptional regulator categorization and analysis tool. *BMC Bioinformatics* **14:** 321.

Du Z, Zhou X, Ling Y, Zhang Z, Su Z. 2010. agriGO: A GO analysis toolkit for the agricultural community. *Nucleic Acids Research* **38:** 64-70.

Dunn CW, Howison M, Zapata F. 2013. Agalma: an automated phylogenomics workflow. *BMC Bioinformatics* **14:** 330.

Enright A J, Von Dongen S, Ouzounis CA. 2002. An efficient algorithm for large-scale detection of protein families. *Nucleic Acids Research* **30:** 1575-1584.

Eshel G, Shaked R, Kazachkova Y, Khan A, Eppel A, Cisneros A, Acuna T, Gutterman Y, Tel-Zur

N, Rachmilevitch S *et al*. 2017. *Anastatica hierochuntica*, an *Arabidopsis* desert relative, is tolerant to multiple abiotic stresses and exhibits species-specific and common stress tolerance strategies with its halophytic relative, *Eutrema* (*Thellungiella*) *salsugineum*. *Frontiers in Plant Science* **7:** 1992.

Grabherr MG, Haas BJ, Yassour M, Levin JZ, Thompson DA, Amit I, Adiconis X, Fan L, Raychowdhury R, Zeng Q *et al*. 2011 Full-length transcriptome assembly from RNA-Seq data without a reference genome. *Nature Biotechnology* **29:** 644-652.

Greenfield P, Duesing K, Papanicolaou A, Bauer DC. 2014. Blue: Correcting sequencing errors using consensus and context. *Bioinformatics* **30:** 2723-2732.

Haas BJ, Papanicolaou A, Yassour M, Grabherr M, Philip D, Bowden J, Couger MB, Eccles D, Li B, Lieber M, *et al*. 2014. *De novo* transcript sequence reconstruction from RNA-seq using the Trinity platform for reference generation and analysis. *Nature Protocols* **8:** 1494-1512.

Hubisz MJ, Pollard KS, Siepel A. 2011. Phastand Rphast: Phylogenetic analysis with space/time models. *Briefings in Bioinformatics* **12:** 41-51.

Katoh K, Kuma KI, Toh H, Miyata T. 2005. MAFFT version 5: Improvement in accuracy of multiple sequence alignment. *Nucleic Acids Research* **33:** 511-518.

Kazachkova Y, Eshel G, Pantha P, Cheeseman JM, Dassanayake M, Barak S. 2018. Halophytism: What have we learnt from *Arabidopsis thaliana* relative model systems? *Plant Physiology* **178:** 972-988.

Kolde R. 2012. Pheatmap: pretty heatmaps, R package v. 16 (R Foundation for Statistical Computing)

Langfelder P, Horvath S. 2008. WGCNA: An R package for weighted correlation network

analysis. *BMC Bioinformatics* **9:** 559.

Langmead B, Trapnell C, Pop M, Salzberg S. 2009. Ultrafast and memory-efficient alignment of short DNA sequences to the human genome. *Genome Biology* **10:** R25.

Li B, Dewey CN. 2011. RSEM: accurate transcript quantification from RNA-Seq data with or without a reference genome. *BMC Bioinformatics* **12:** 323.

Livak KJ, Schmittgen TD. 2001. Analysis of relative gene expression data using real-time quantitative PCR and the $2^{-\Delta\Delta C}{}_T$ method. *Methods* **25:** 402-408.

Love MI, Huber W, Anders S. 2014. Moderated estimation of fold change and dispersion for RNA-seq data with DESeq2. *Genome Biology* **15:** 550.

Maere S, Heymans K, Kuiper M. 2005. BiNGO: a Cytoscape plugin to assess overrepresentation of gene ontology categories in biological networks. *Bioinformatics* **21:** 3448-3449.

Margulies M, Egholm M, Altman WE, Attiya S, Bader JS, Bemben LA, Berka J, Braverman MS, Chen Y-J, Chen Z *et al*. 2005. Genome sequencing in microfabricated high-density picolitre reactors. *Nature* **437:** 376-380.

Moriya Y, Itoh M, Okuda S, Yoshizawa AC, Kanehisa M. 2007. KAAS: An automatic genome annotation and pathway reconstruction server. *Nucleic Acids Research* **35:** (Web Server issue) W182-W185.

Murashige T, Skoog F. 1962. A revised medium for rapid growth and bioassays with tobacco tissue cultures. *Physiologia Plantarum* **15:** 473-497.

Panwar B, Arora A, Raghava GP. 2014. Prediction and classification of ncRNAs using structural information. *BMC Genomics* **15:** 127.

Rio DC, Ares Jr M, Hannon GJ, Nilsen TW. 2010. Purification of RNA Using TRIzol (TRI Reagent).

Cold Spring Harbor Protocols doi:10.1101/pdb.prot5439.

Schmieder R, Edwards R. 2011. Fast identification and removal of sequence contamination from genomic and metagenomic datasets. *PloS One* **6:** e17288

Shannon P, Markiel A, Ozier O, Baliga NS, Wang JT, Ramage D, Amin N, Schwikowski B, Ideker T. 2003. Cytoscape: a software environment for integrated models of biomolecular interaction networks. *Genome Research* **13:** 2498-2504.

Simão FA Waterhouse RM, Ioannidis P, Kriventseva EV, Zdobnov EM. 2015. BUSCO: assessing genome assembly and annotation completeness with single-copy orthologs. *Bioinformatics* **31:** 3210-3212.

Stamatakis A. 2014. RAxML version 8: A tool for phylogenetic analysis and post-analysis of large phylogenies. *Bioinformatics* **30:** 1312-1313.

Suyama M, Torrents D, Bork P. 2006. PAL2NAL: Robust conversion of protein sequence alignments into the corresponding codon alignments. *Nucleic Acids Research* **34:** (Web Server Issue) W609-612.

Szcześniak MW, Rosikiewicz W, Makałowska I. 2015 CANTATAdb: a collection of plant long non-coding RNAs. *Plant & Cell Physiology* **57:** e8.

Wang G, Oh DH Dassanayake M. 2020. GOMCL: a toolkit to cluster, evaluate, and extract non-redundant associations of Gene Ontology-based functions. *BMC Bioinformatics* **21:** 139.

Whelan S, Goldman N. 2001. A general empirical model of protein evolution derived from multiple protein families using a maximum-likelihood approach. *Molecular Biology & Evolution* **18:** 691-699.

Yang ZH. 1997. PAML: a program package for phylogenetic analysis by maximum likelihood.

Computer Applications in the Biosciences **13:** 555-556.

Yang Z. 2007. PAML 4: Phylogenetic analysis by maximum likelihood. *Molecular Biology & Evolution* **24:** 1586-1591.

Zhang M, Leong HW. 2010. Bidirectional best hit *r*-window gene clusters. BMC Bioinformatics **11:** S63.

Zhao Y, Li H, Fang S, Kang Y, Wu W, Hao Y, Li Z, Bu D, Sun N, Zhang MQ, *et al*. 2015. NONCODE 2016: an informative and valuable data source of long non-coding RNAs. *Nucleic Acids Research* **44:** (D1) D203-D208.

Zhou Y, Zhu J, Tong T, Wang J, Lin B and Zhang J. 2019. A statistical normalization method and differential expression analysis for RNA-seq data between different species. *BMC Bioinformatics* **20:** 163.
